# Trametinib activates endogenous neurogenesis and recovers neuropathology in a model of Alzheimer’s disease

**DOI:** 10.1038/s12276-023-01073-2

**Published:** 2023-10-02

**Authors:** Mi-Yeon Kim, Mi Jeong Kim, Changyeob Lee, Juwon Lee, Sang Seong Kim, Sungho Hong, Hyoung Tae Kim, Jinsoo Seo, Ki-Jun Yoon, Sungho Han

**Affiliations:** 1 Neuroscience Research Center, Genuv Inc., Seoul, 03175 Republic of Korea; 2 Department of Brain Sciences, Daegu Gyeongbuk Institute of Science and Technology (DGIST), Daegu, 42988 Republic of Korea; 3 College of Pharmacy, Hanyang University ERICA, Gyeonggi-do, 15588 Republic of Korea; 4 Computational Neuroscience Unit, Okinawa Institute of Science and Technology, Okinawa, 904-0495 Japan; 5 Department of Biological Sciences, Korea Advanced Institute of Science and Technology (KAIST), Daejeon, 34141 Republic of Korea; 6 KAIST Stem Cell Center, KAIST, Daejeon, 34141 Republic of Korea; 7 Head Office, Genuv Inc., Seoul, 04520 Republic of Korea; 8 Genuv US Subsidiary, Genuv Inc., Cambridge, USA; 9 Present Address: Department of Biomedical Science and Engineering, Gwangju Institute of Science and Technology, Gwangju, 61005 Republic of Korea; 10 Present Address: Shaperon Inc., Seoul, 06373 Republic of Korea

**Keywords:** Alzheimer's disease, Adult neurogenesis

## Abstract

Enhancing adult neurogenesis in the brain has been suggested as a potential therapeutic strategy for AD. We developed a screening platform, ATRIVIEW^®^, for molecules that activate neuronal differentiation of adult mouse NSCs. The most potent hit from an FDA-approved drug library was SNR1611 (trametinib), a selective MEK1/2 inhibitor. We found that trametinib increases the levels of P15^INK4b^ and Neurog2, suggesting a mechanism by which MEK1/2 inhibition induces neuronal differentiation. Oral administration of trametinib increased adult neurogenesis in the dentate gyrus and subventricular zone of the 5XFAD AD mouse model. Surprisingly, we also found that trametinib enhanced adult neurogenesis in the cortex. Consequently, trametinib rescued AD pathologies such as neuronal loss and cognitive impairment in 5XFAD mice. Finally, trametinib induced neurogenic differentiation of NSCs derived from AD patient iPSCs, which suggests its potential therapeutic application. Altogether, we suggest that restoration of endogenous adult neurogenesis by trametinib may be a promising therapeutic approach to AD.

## Trametinib: A Surprising Key in Alzheimer’s Disease Battle

“Trametinib, a MEK1/2 inhibitor, promotes adult neurogenesis in an Alzheimer’s disease mouse model and human iPSC-derived neural stem cells." Researchers have discovered that trametinib, a MEK1/2 inhibitor, can enhance adult neurogenesis and replenish neurons in brain areas where neurodegeneration most affects Alzheimer’s disease (AD). The study found that trametinib induced neuronal differentiation of adult neural stem cells and restored impaired neurogenesis in the hippocampus and subventricular zone of the AD model mice, in addition to inducing adult cortical neurogenesis. It also demonstrated trametinib’s potential for inducing neurogenic differentiation in AD patient-induced pluripotent stem cells (iPSCs)-derived NSCs. These findings suggest that trametinib could be a promising therapeutic approach for treating AD and other neurodegenerative diseases by promoting adult neurogenesis.

## Introduction

Alzheimer’s disease (AD) is characterized by impaired cognitive functions and memory loss caused by neuronal degeneration in the cortex and hippocampus. Pathogenic mechanisms of AD have not been clearly elucidated, but it is known that deposition of amyloid plaques or hyperphosphorylated tau tangles damages neuronal networks through diverse events such as autophagic-lysosomal dysfunction, mitochondrial dysregulation, synaptic loss, and neuroinflammation^1,2^. Thus, the development of AD treatments has mainly focused on eliminating amyloid plaques and tau tangles. However, since AD patients have already undergone significant neuronal damage, such disease-modifying therapies have been unsuccessful. Recently, regenerative approaches, including stem cell therapy, have attracted attention as a treatment for neurodegenerative diseases such as Parkinson’s disease and AD^3^. Unfortunately, these approaches also have many challenges, such as securing a sufficient amount of stem cells to be administered to the patient, the risk of tumor formation caused by a viral infection of stem cells, the difficulty in technology of differentiating stem cells to the required cell type, and the problem of immune rejection^4^.

Adult neurogenesis has been observed in the subventricular zone (SVZ) of the lateral ventricle and the subgranular zone (SGZ) of the hippocampal dentate gyrus. Several studies have also reported evidence of adult neurogenesis in the neocortex, striatum, amygdala, hypothalamus, substantia nigra, and brain stem^5,6^. Recently, it was reported that neurogenesis in the adult brain, especially in the hippocampus, is maintained until 90 years of age, but it decreases sharply in AD patients^7,8^. Compared with healthy individuals, the number of neural progenitor cells (NPCs) in the SVZ and SGZ in the brains of AD patients is abnormally increased^9^. At the same time, however, the number of migrating neuroblasts and newly differentiated mature neurons decreased due to the slowed progression of essential processes in neurogenesis along with disease progression^8^. In addition, profound impairment of adult hippocampal neurogenesis could be observed from the early stage of AD^8^. Therefore, restoration of endogenous adult neurogenesis may be a promising therapeutic approach for AD.

The mitogen-activated protein kinase kinase (MEK) and extracellular signal-related kinase (ERK) signaling pathways engage in numerous physiological processes, including proliferation, differentiation, survival, and death of various cell types^10–12^. Activation of the MEK/ERK pathway inhibits neuronal differentiation in developing brains^13–15^, whereas inhibition of the same signaling induces differentiation of NSCs^16,17^. In addition, Aβ oligomers inhibit NSC migration in the neurosphere assay through MEK/ERK signaling activation^18^. Therefore, MEK/ERK signaling has been considered an essential modulator of neurogenesis.

Here, we report that trametinib, a specific MEK1/2 inhibitor, is identified by the phenotype-based screening platform ATRIVIEW^®^ to induce neuronal differentiation of adult NSCs from an AD mouse model. Trametinib treatment of NSCs induces the expression of the cell cycle inhibitor P15^INK4b^^19^ and proneuronal factor Neurog2^20^ in vitro. By inhibiting MEK/ERK signaling, trametinib promotes adult neurogenesis of NSCs in the SVZ, DG, and cortex of 5XFAD AD model mice. Trametinib also rescues neuronal cell number and axonal length in the brain and recovers cognitive functions in AD model mice. Moreover, we provide evidence that trametinib can induce neurogenic differentiation of NSCs derived from AD patient iPSCs.

## Materials and methods

### Animals

B6SJL-Tg (APPSwFlLon,PSEN1*M146L*L286V) (5XFAD) mice and their age-matched wild-type (WT) mice (B6SJLF1/J) were purchased from The Jackson Laboratory, and experimental procedures were performed according to protocols approved by the Institutional Animal Care and Use Committee (IACUC) of KPCLab (approved number: P171011), MEDIFRON DBT Inc. (approved number: Medifron 2017–1), Korea Brain Research Institute (approved number: IACUC-19–00042) and WOOJUNG BIO (approved number: IACUC2004–046).

### Trametinib treatment

Trametinib (MedChemExpress, Monmouth Junction, NJ) was micronized and suspended in vehicle containing 5% mannitol, 1.5% hydroxypropyl methylcellulose, and 0.2% sodium lauryl sulfate. 5XFAD mice and their age-matched WT mice (male, *n* = 7 ~ 10 per group) were divided into vehicle- and trametinib-treated groups. Vehicle or 0.1 mg/kg trametinib was administered to mice in each group for 1, 1.5 or 2.5 months by oral gavage once a day. Temozolomide (TMZ; MedChemExpress, Monmouth Junction, NJ) was dissolved in 0.9% NaCl. Seven-month-old 5XFAD mice and their age-matched WT mice were divided into vehicle-, trametinib-, TMZ-, and trametinib/TMZ-treated groups. Twenty-five mg/kg TMZ was injected into mice intraperitoneally, and the animals were injected on 3 consecutive days every week for a total of 6 weeks. All mice were sacrificed by the perfusion method, and brain samples were processed for biochemical and immunohistochemical analyses. The pharmacokinetic and pharmacodynamic profiles of trametinib in the blood and brain were previously described^21^.

### Behavior tests

#### Novel object recognition test

To test novel object recognition, mice were habituated in an empty open field arena (42 cm × 42 cm). For the training trial, mice were placed in an open field arena with two identical objects for 5 min each. The next day, the test trial was performed for 5 min with one of the two familiar objects replaced with a new one. Video tracking was performed, and the time that mice explored the novel object and the time that mice explored the familiar object were measured. The discrimination index (DI) was defined as (novel object – familiar object)/(novel object + familiar object).

#### Fear conditioning test

For the training test, mice were placed into a fear chamber. After allowing them to move freely for 200 s in a darkroom box (fear chamber), a sound of 460 Hz, 75 db was delivered for 5 s, and stimulation was given with a current of 0.5 mA strength for 2 s immediately after the sound. These sounds and electrical stimulations were repeated a total of 4 times. For the contextual fear test, 24 h after the training test, the mice were placed in the fear chamber, and contextual fear was measured for 120 s. Freezing behavior was analyzed every 60 sec to report the percentage of freezing.

### Cell culture

#### Adult neural stem cells (NSCs)

Primary NSCs were isolated from the subventricular zone (SVZ) of 8-week-old C57BL/6 mice or 8-month-old 5XFAD mice, and neurospheres were cultured as previously described^22,23^. For neurosphere cultures from normal mice or 5XFAD mice, cells were dissociated from brain tissue and grown in DMEM/F12 medium contining N2 supplement in 25 cm^2^ flasks in suspension. Twenty nanograms/ml basic FGF (Peprotech, 100–18B-100) and 20 ng/ml human EGF (Peprotech, AF-100–15–500) were added to the medium to allow the cells to form neurospheres. For analyses, NSCs were cultured as a monolayer. For differentiation of NSCs, the neurospheres were dissociated with 0.025% trypsin-EDTA (Invitrogen, 15400054), plated on poly-L-ornithine (10 μg/ml, Sigma‒Aldrich, P2533)/laminin (5 μg/ml, Roche, 11243217001)-coated plates, and cultured in bFGF- and EGF-depleted DMEM/F12 medium containing N2 supplement for 2 days.

For transient transfection, NSCs were cultured for 1 day and then transfected with the required plasmids using Lipofectamine 2000 transfection reagent (Thermo Fisher, cat. no. 11668019) according to the manufacturer’s instructions. For MEK knockdown experiments, NSCs were transfected with shRNA-MEK1 and shRNA-MEK2 using Lipofectamine. After 4 h of incubation, cells were further grown in growth factor-depleted medium. After 48 h, the cells were harvested. Mouse Map2k1 #: GAGGCCTCTCAGCTCATAT (Dharmacon V3SM11241–234594572) and mouse Map2k2 #1: ACATGTCCCCAGAGCGGCT (Dharmacon V3SM11241–235104473) were used.

#### Human-induced pluripotent stem cells (hiPSCs)

Human iPSC lines from a patient with familial Alzheimer’s disease (PSEN1 M146I) and from a healthy individual were purchased from the Coriell Institute (AD patient: #ND34732/female/Age 33, healthy individual: #GM23720/female/Age 22). Another healthy human iPSC line was obtained from Dr. Bruce Yankner’s laboratory at Harvard Medical School, in which stemness, pluripotency, and normal karyotypes were certified^24^. One day prior to seeding iPSCs, 0.1% gelatin solution was coated on a six-well plate and incubated for 2 h at 37 °C. To support the adhesion of iPSCs, mouse embryonic fibroblasts (MEFs, Gibco) were seeded in advance as a feeder cell and incubated overnight with MEF media [DMEM, high glucose (Gibco), 10% FBS (Gibco), 1x nonessential amino acid (NEAA, Thermo Fisher Scientific), 1x GlutaMAX (Thermo Fisher Scientific), 1x sodium pyruvate (Thermo Fisher Scientific)]. iPSCs were maintained in human embryonic stem cell (hES) media [20% Knock-out serum replacement (KSR, Gibco), 1x NEAA (Gibco), 1x GlutaMAX (Thermo Fisher Scientific), 12 nM FGF2 (Peprotech) and 0.1 mM 2-mercaptoethanol (Sigma‒Aldrich) in DMEM/F12, HEPES media (Gibco)]. When iPSCs were stabilized, cells were transferred to a 6-well plate precoated with hESC-qualified Matrigel (Corning) for at least 2 hr in advance. On the Matrigel plate, iPSCs were fed with mTeSR1 media (STEMCELL Technologies) every single day.

#### Neural stem cell differentiation from hiPSCs

iPSCs were cultured in mTeSR1 media until they reached 100% confluence. Then, cells were differentiated into NSCs by replacing the media with neuronal induction media [DMEM/F-12 GlutaMAX (Gibco), Neurobasal (Gibco), 0.5x N2 (Gibco), 0.5x B27 (Gibco), 0.5x GlutaMAX (Thermo Fisher Scientific), 5 μg/ml insulin (Sigma‒Aldrich), 0.5x NEAA (Thermo Fisher Scientific), 100 μM 2-mercaptoethanol (Sigma‒Aldrich), 1x Penicillin/Streptomycin (Gibco)] with 1 μM Dorsomorphin (Tocris) and 10 μM SB431542 (Tocris) for 11 days. Then, the cells were transferred to a new Matrigel-coated six-well plate and fed without dorsomorphin and SB431542. When the neural rosette was apparent, cells were transferred to a new plate at a density of 3 × 10^5^ cells/cm^2^ and fed neuronal maintenance media (neuronal induction media with 20 ng/ml FGF2).

### Drug screening

For ATRIVIEW^®^, we used NSCs from Tg2576 mice, which were kindly provided by Dr. Manho Kim at Seoul National University Hospital. For neurosphere cultures from Tg2576 mice, cells were grown in DMEM/F12 with B27 supplements in 75 cm^2^ flasks in suspension. bFGF (20 ng/ml) and human EGF (20 ng/ml) were added to the media to allow the cells to form neurospheres. In growth factor-free medium, Tg2576-derived NSCs start to deposit Aβ^23^. For drug screening, adult NSCs derived from Tg2576 were plated on poly-L-ornithine/laminin-coated 96-well plates. After 24 h, the cells were treated with 500 nM FDA-approved drugs (994 compounds) and incubated in growth factor-free medium for 48 h. Immunocytochemistry was performed using an anti-Tuj1 antibody (Cell Signaling, 4466; 1:200), which is a neuron-specific marker^25^, and Alexa Fluor 488 (Thermo, A21121; 1:200). The nuclei were stained with 4’,6-diamidino-2-phenylindole (DAPI). Fluorescence was measured using a Varioskan Flach fluorometer (Thermo Fisher, VLBL00D0 N12391).

### Immunocytochemistry

NSCs and iPSC-derived NSCs were cultured on glass coverslips. After washing three times with PBS for 5 min, the cells were fixed in 10% formalin for 10 min at room temperature. The cells were then washed with PBS and permeabilized in 0.1% Triton X-100 for 15 min. Cells were placed in blocking solution containing 5% BSA and 10% normal goat serum (NGS) in PBS for 1 h at room temperature and then incubated with primary antibodies in 1% BSA and 10% NGS in PBS overnight. After washing, the cells were incubated with fluorescence-conjugated secondary antibodies for 1 hr at room temperature. After washing, the cells were incubated with 4’,6-diamidino-2-phenylindole (DAPI) for 5 min. Coverslips were mounted using mounting medium (Biomeda, M01) and visualized by confocal microscopy using an LSM880 microscope (Carl Zeiss). Antibodies are indicated in Supplementary Table 2.

### Western blotting

Cultured cells and mouse brain tissues were washed twice with ice-cold phosphate-buffered saline (PBS) and extracted by homogenizing with RIPA buffer (50 mM Tris-HCl, 150 mM NaCl, 0.25% deoxycholic acid, 1% NP-40, 1 mM EDTA, and protease inhibitors, pH 7.4). Lysates were centrifuged at 13,000 rpm for 20 min at 4 °C, and the protein concentration in the supernatant was determined using the Bradford assay (Bio-Rad, 5000006). Proteins from each sample were subjected to 8% ~ 15% SDS‒PAGE, and the resolved proteins were transferred to nitrocellulose membranes. The membranes were blocked with 5% nonfat milk powder in Tris-buffered saline/Tween 20 (TBST) for 1 h at room temperature and then incubated with primary antibodies overnight at 4 °C. After washing, the membranes were incubated with secondary antibodies for 1 h at room temperature. Peroxidase activity was visualized with enhanced chemiluminescence. The detected signals were quantified using an iBright^TM^ imaging system (Thermo Fisher, A44115). Antibodies are indicated in Supplementary Table 2.

### Quantitative PCR (qRT‒PCR)

Quantitative PCR analysis was performed as previously described^26^. Total RNA was extracted from cells using TRIzol (Invitrogen, 15596018). Reverse transcription was performed using M-MLV reverse transcriptase (Thermo Fisher, 28025013). qRT‒PCR was performed using the SYBR^TM^ Green PCR master mix (Thermo Fisher, 4368706) according to the manufacturer’s guidelines. The results were expressed relative to the housekeeping gene *GAPDH* (glyceraldehyde-3-phosphate dehydrogenase). To analyze the expression of mouse or human genes, the primers are indicated in Supplementary Tables 3 and 4.

### Immunohistochemical analysis

Mice were perfused with ice-cold phosphate-buffered saline (PBS) followed by 4% paraformaldehyde (PFA). Brains were dissected and analyzed by immunohistochemistry^27^. For paraffin sections, brain hemispheres were embedded in paraffin and prepared into 5 μm sagittal sections. Paraffin sections were deparaffinized, and antigen retrieval was performed in citrate buffer (pH 6.0). For cryosections, brain hemispheres were embedded in O.C. compound and prepared into 10 μm sagittal sections. The cryosections were air dried before use. For immunostaining, the sections were incubated with primary antibodies overnight at 4 °C. This step was followed by incubation with secondary antibodies for 1 h at room temperature. The sections were counterstained with DAPI. Immunofluorescence images were captured using an LSM880 Laser-Scanning confocal microscope (Carl Zeiss). For diaminobenzidine (DAB) staining, immunohistochemistry was performed with the DAB kit (Vector Lab., SK4100). Immunostained cells were counted in the area using the Icy micromanager program (Institut Pasteur) or Zen software (Carl Zeiss). Cortical neurons were counted in the somatosensory cortex of 5XFAD mice.

### Whole-cell RNA sequencing

RNA was isolated from mouse whole brains, and cDNA libraries for RNA sequencing were prepared using the TruSeq Stranded mRNA Prep Kit (Illumina, San Diego, CA) according to the manufacturer’s guidelines^28^. The libraries were sequenced on the Illumina Nextseq500 platform, and the reads were mapped to the reference mouse mm10 genome using TopHat v2.0.13. The total number of reads mapped to the transcriptomes was 24,532 genes, and the genes with a count of 0 in at least one sample were removed before differential expression analysis. There were a total of 15,727 genes after the removal of genes with a count of 0. To define differentially expressed genes (DEGs), we set up a stringent statistical cutoff of a fold change (FC) ≥ 1.5 and a false discovery rate (FDR) < 0.05. A total of 160 DEGs (107 genes were upregulated and 53 genes were downregulated) were identified between the vehicle-treated group and trametinib-treated group in the second week. Gene ontology was performed with the biological process using the Panther database. The significance threshold for analyses was set to 0.05 using Fisher’s exact test-adjusted *p* values.

### Statistical analysis

All data were analyzed in Prism (GraphPad Software). All graphical data are presented as the mean ± s.e.m. Data distribution was assumed to be normal, and this was not formally tested. Mice were randomly assigned to experimental groups, and no animals or data points were excluded from analyses. Investigators were blinded to group allocation during data collection and analysis. No statistical methods were used to predetermine sample sizes; sample sizes were determined on the basis of pilot studies, and randomization procedures were not used. Statistical differences between groups were determined using one-tailed unpaired Student’s t test or one-way ANOVA followed by Dunnett’s analysis or Fisher’s LSD analysis. *P* values and degrees of freedom are described in the figure legends. Significance was reported as *P < 0.05, **P < 0.005 or ***P < 0.001.

## Results

### ATRIVIEW^®^, the CNS regenerative drug screening platform, and properties of its hit trametinib (SNR1611)

**Fig. 1:**
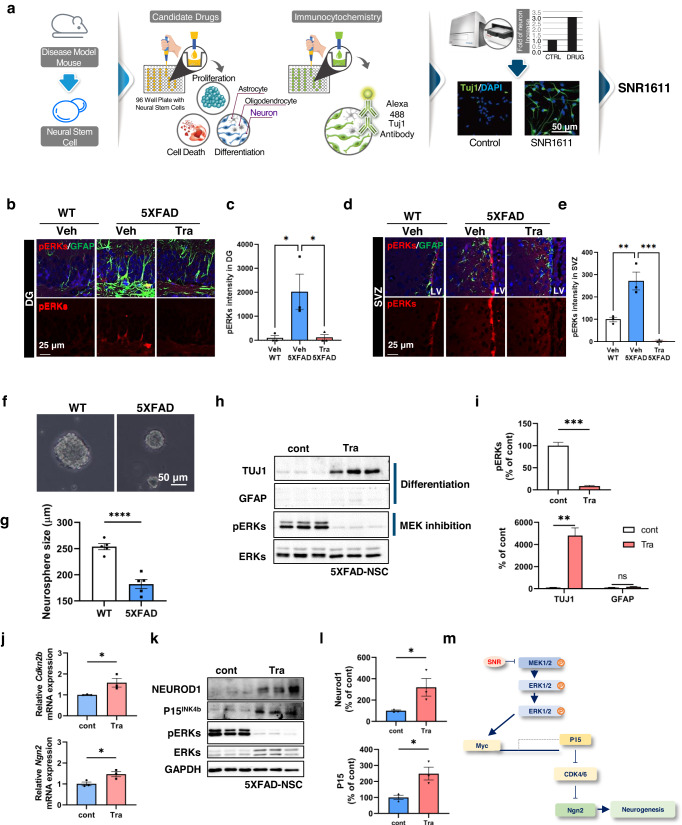
Identification and characterization of trametinib (SNR1611), a small molecule that induces neuronal differentiation of adult NSCs from 5XFAD mice. **a** Schematic diagram of drug screening using the ATRIVIEW^®^ platform. Changes in the morphology and Tuj1 expression level of adult NSCs from Tg2576 AD model mouse brains upon treatment with FDA-approved drugs were monitored. **b**–**e** MEK/ERK signaling was activated in GFAP-positive cells in the hippocampal DG (**b**, **c**) and SVZ (**d**, **e**) of 5XFAD mice. Immunofluorescence staining images (**b**, **d**) and quantification of pERK intensities (**c**, **e**) in the hippocampal DG or SVZ of 7.5-month-old 5XFAD mice. Trametinib was administered to 5-month-old 5XFAD mice for 2.5 months. *n* = 3 mice per group; sagittal sections from each mouse brain. Scale bars, 25 μm. Normalized to the WT-vehicle group. **f**, **g** Adult NSCs from 5XFAD mice were cultured to form neurospheres in a medium containing 20 ng/ml EGF and 20 ng/ml bFGF. Gross images were obtained on the 4th day after seeding (**f**), and neurosphere size was quantified (**g**). Scale bars, 50 μm. **h**–**l** Adult NSCs from 5XFAD mice were seeded on plates and treated with 100 nM trametinib in medium without growth factors for 48 h. Cell lysates were subjected to immunoblot analyses of TUJ1, GFAP, pERKs and ERKs (**h**) and quantified by band intensities (**i**). The levels of *Cdkn2b* and *Ngn2* mRNA were analyzed using qRT‒PCR. Each sample was normalized to the expression of *Gapdh* (**j**). Cell lysates were subjected to immunoblot analyses of P15 and Neurod1 (**k**), and band intensities were quantified (**l**). **m** The proposed mechanism by which MEK1/2 inhibition induces cell cycle arrest and neuronal differentiation. Data are representative of three independent experiments, and values are expressed as the mean ± SEM. *P* values were obtained by one-way ANOVA (**c**, **e**) and Student’s *t*-test (**i**–**l**). *p < 0.05, **p < 0.005 and ***p < 0.001.

Because drugs against AD aiming for Aβ clearance or to interfere with Aβ production have not been successful in restoring already damaged cognitive functions, we hypothesized that enhancing adult neurogenesis from endogenous NSCs will restore neuronal network integrity in affected brain regions of AD patients. Thus, we developed a phenotypic drug discovery platform, ATRIVIEW^®^, aiming to identify small molecular compounds inducing neuronal differentiation of adult NSCs derived from AD model mice (AD-NSCs) (Fig. 1a). Using NSCs derived from the Tg2576 AD mouse model, we screened 994 small molecules from an FDA-approved drug library to identify small molecules inducing neuronal differentiation. Upon comparing the level of Tuj1 (class III beta-tubulin, neuronal marker) expression using fluorescent immunocytochemical analysis, 48 compounds were found to increase neuronal differentiation (at least 1.7-fold increase compared to the DMSO-treated control) (Supplementary Fig. 1a). Among them, the most effective drug inducing neuronal differentiation of NSCs was SNR1611, which was trametinib (Mekinist®), a specific MEK1/2 inhibitor and FDA-approved anticancer drug. Upon immunocytochemistry, it was observed that trametinib treatment induced morphological changes of NSCs into neuron-like cells (Fig. 1a). Another MEK1 inhibitor, cobimetinib^29^, and a CDK inhibitor, dinaciclib^30^, were among the selected drugs. Semagacestat^31^, a g-secretase inhibitor, and duloxetine^32^, a serotonin-norepinephrine reuptake inhibitor, which are known for their therapeutic potential for AD, were also screened (Supplementary Fig. 1a). In the presence of growth factors in the medium (20 ng/ml EGF and 20 ng/ml bFGF), trametinib inhibited proliferation (assessed by PCNA level) and induced neuronal differentiation (by Tuj1 level) but not astrocytic differentiation (by GFAP level) of adult NSCs from C57B/L6 mice (Supplementary Fig. 1b). Regardless of the presence of the Aβ_1–42_ oligomer (10 mM), both MEK1/2 inhibition by trametinib (Supplementary Fig. 1c) and MEK1/2 knockdown (Supplementary Fig. 1d) induced neuronal differentiation of NSCs. These findings indicate that inhibition of MEK/ERK signaling by trametinib is a potential approach to induce neuronal differentiation of adult NSCs.

### Inhibition of MEK/ERK signaling by trametinib induces neuronal differentiation via induction of *P15*^*INK4b*^ and *Neurog2* expression and protects against cell death

It has been reported that MEK/ERK signaling is activated in the brain of AD patients compared to the normal brain^33,34^, and Aβ plaques activate this signaling^35^. To determine whether MEK/ERK signaling is also activated in NSCs of AD model mice with Aβ plaque phenotypes, we measured the pERK level in the brains of WT and 5XFAD AD model mice. It was confirmed that the MEK/ERK signaling pathway was activated in the SGZ (Fig. 1b, c) and SVZ (Fig. 1d, e) of 7.5-month-old 5XFAD mice compared with WT mice. Administration of trametinib (for 2.5 months to 5-month-old 5XFAD mice) reduced the level of pERKs in both areas (Fig. 1b–e). Specifically, pERK-positive NSCs, confirmed as Nestin-positive cells, were increased in 5XFAD mice and decreased by trametinib administration (Supplementary Fig. 2). The level of pERKs, the downstream substrate of MEK1/2, was also increased in the cortex of 5XFAD mice compared to WT mice and was decreased by trametinib administration (Supplementary Fig. 3). These results confirmed that trametinib penetrates the brains of 5XFAD mice and inhibits MEK-ERK signaling. The bioavailability of trametinib in the brain has also been reported in a previous pharmacokinetics (PK) study^21^. The brain PK profile indicated good penetration of trametinib into the mouse brain, where the brain-plasma ratio (B/P ratio) was measured to be ~0.4–0.6^21^. We further tested whether trametinib induces neuronal differentiation of adult NSCs isolated from 5XFAD mice. The neurospheres derived from 5XFAD mice were smaller than those from wild-type (WT) mice (Fig. 1f, g). Trametinib strongly increased the level of Tuj1 but not the level of GFAP in NSCs isolated from 5XFAD mice, indicating its induction of neuronal but not astrocytic differentiation (Fig. 1h, i). In addition, we confirmed that other MEK1/2 inhibitors (AZD8330, PD184352, Refametinib, PD318088, AS703026) also induced neuronal differentiation (Supplementary Fig. 4) in embryonic NSCs, indicating that MEK1/2 inhibition activates neuronal differentiation. Notably, we observed that trametinib was the most effective MEK inhibitor to induce neuronal differentiation.

Since trametinib was discovered as a drug to induce CDK inhibitor (such as P15^INK4b^)-mediated cell cycle arrest by inhibiting MEK1/2^36^, we tested whether it would induce neuronal differentiation through cell cycle arrest in NSCs as well. We found that trametinib induced P15^INK4b^ (cell cycle arrest) and Neurog2 (proneuronal factors) expression and increased the protein levels of P15^INK4b^ and Neurod 1 (neuronal differentiation 1) in adult NSCs from 5XFAD mice (Fig. 1j–m). Whole brain RNA-Seq analysis also supported the concept that inhibition of MEK/ERK signaling is the mechanism responsible for neuronal differentiation. In GO term analysis, the second week of trametinib administration appeared to be the critical period for neuron development, pyramidal neuron differentiation, and regulation of neuron migration (Supplementary Fig. 5). Among the 107 genes whose expression was increased by 1.5 times or more, the expression of the *Ebf1, Nhlh2, Irx5, Ebf3, Irx3, Sox14, Cdh1*, and *Tead4* genes, known as the target genes of *Ngn2*, was increased by trametinib administration (Supplementary Table 1).

To investigate whether apoptosis was induced by Aβ accumulation as in previous results^23^ in adult NSCs isolated from 5XFAD mice and whether trametinib protects against this accumulation, we examined the expression of apoptosis markers. Aβ accumulation was observed in adult NSCs from 5XFAD mice, as was active caspase-3. Trametinib treatment, however, reduced the level of Aβ accumulation and active caspase-3. In addition, the level of cleaved poly (ADP-ribose) polymerase (PARP), another apoptosis marker, was decreased by trametinib treatment (Supplementary Fig. 6a–e). MEK1/2 knockdown by shRNA also reduced the expression of active caspase 3 (Supplementary Fig. 6f, g).

Taken together, these results demonstrate that activation of MEK/ERK signaling is related to the apoptosis of adult NSCs of 5XFAD mice and that trametinib protects against cell death. Furthermore, trametinib enhanced neuronal differentiation through the induction of CDK inhibitors and proneuronal factors.

### Trametinib induces hippocampal and SVZ neurogenesis in 5XFAD mice

**Fig. 2:**
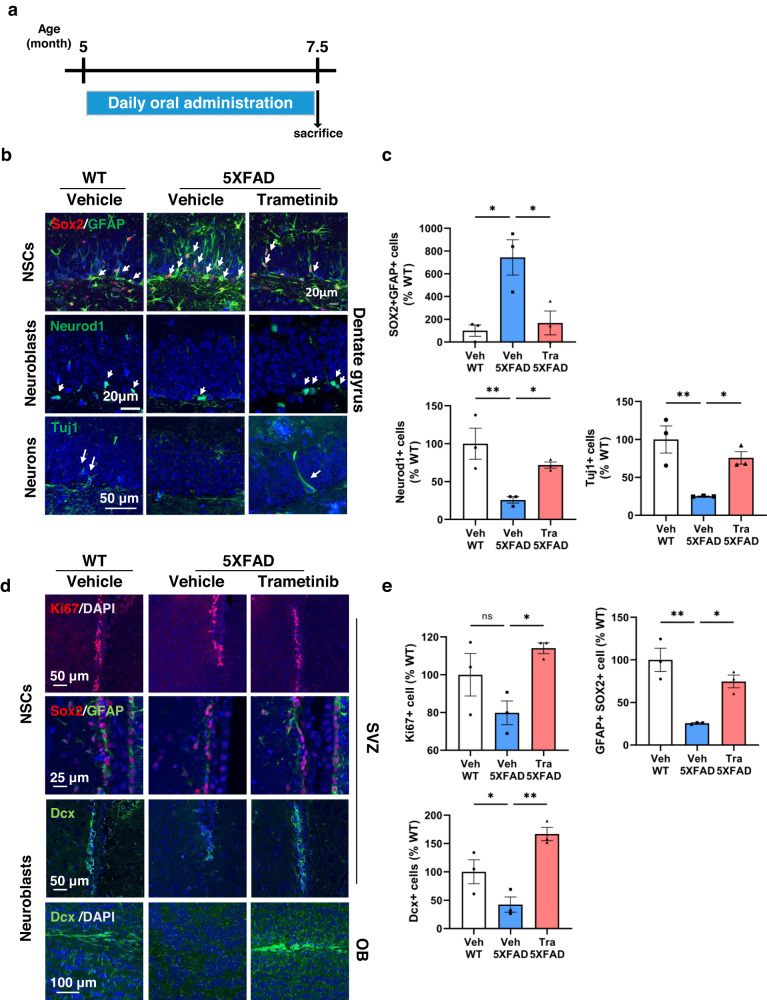
Trametinib induces adult neurogenesis in the 5XFAD mouse brain. **a** Scheme of trametinib administration to 5XFAD mice. Trametinib was administered to 5-month-old 5XFAD mice for 2.5 months. **b**, **c** Trametinib administration induced adult neurogenesis in the hippocampal DG. Immunofluorescence staining images (**b**) and quantification of stained cell numbers (**c**) in the hippocampal DG of 7.5-month-old 5XFAD mice. Arrows indicate stained cells. *n* = 3 mice per group; sagittal sections from each mouse brain. Scale bars, 20 μm or 50 μm. Normalized to the WT-vehicle group. **d**, **e** Trametinib administration induced adult neurogenesis in the SVZ. Immunofluorescence staining images (**d**) and quantification of stained cell number (**e**) in the SVZ or olfactory bulb (OB) of 7.5-month-old 5XFAD mice. Arrows indicate stained cells. *n* = 3 mice per group; sagittal sections from each mouse brain. Scale bars, 25 μm, 50 μm or 100 μm. Normalized to the WT-vehicle group. Data are representative of three independent experiments, and values are expressed as the mean ± SEM. *P* values were obtained by one-way ANOVA. *p < 0.05 and **p < 0.005.

Since trametinib induced neuronal differentiation of NSCs derived from AD model mice, we examined whether oral administration of trametinib also induces hippocampal neurogenesis in 5XFAD mice. Five-month-old 5XFAD mice were administered 0.1 mg/kg trametinib for 2.5 months, after which all mice were sacrificed at the age of 7.5 months (Fig. 2a). In the DG of the vehicle-administered 5XFAD mice, Sox2+/GFAP+ NSCs^37^ increased, but the number of both Neurod1+ neuroblasts and Tuj1+ immature neurons decreased compared with the WT control (Fig. 2b, c). In contrast, the number of neuroblasts and immature neurons in the same brain region of the trametinib-administered 5XFAD mice increased, while the number of radial glial cells decreased (Fig. 2b, c).

Next, we asked whether trametinib also induces neuronal differentiation of NSCs in the SVZ of 5XFAD mice^38^. The number of NSCs expressing Sox2 and GFAP (type B1 cells) in the SVZ of 5XFAD mice was decreased compared with that in WT control mice (Fig. 2d, e). In addition, the number of Dcx+ neuroblasts (Type A cells) significantly decreased in 5XFAD mice. Trametinib increased the number of Sox2+/GFAP+ NSCs, Ki67+ proliferating cells and Dcx+ neuroblasts in the SVZ of 5XFAD mice to a level comparable to that observed in WT mice (Fig. 2d, e). These data indicate that the generation of type A neuroblasts from NSCs is impaired in 5XFAD mice and that trametinib restores the transition to facilitate adult neurogenesis in the SVZ.

Then, we asked further whether trametinib induced the migration of newly formed neuroblasts in the SVZ to the olfactory bulb (OB). While the number of Dcx+ neuroblasts was significantly reduced in the OB granular layer of 5XFAD mice, trametinib indeed recovered the neuroblast population (Fig. 2d). These data demonstrate that inducing adult neurogenesis in the SVZ by trametinib restores the migration of neuroblasts to the OB, which is disrupted in AD conditions. Altogether, trametinib administration evidently restores adult hippocampal and SVZ neurogenesis, which is impaired in 5XFAD mice.

### Trametinib induces cortical neurogenesis in the 5XFAD mouse brain

**Fig. 3:**
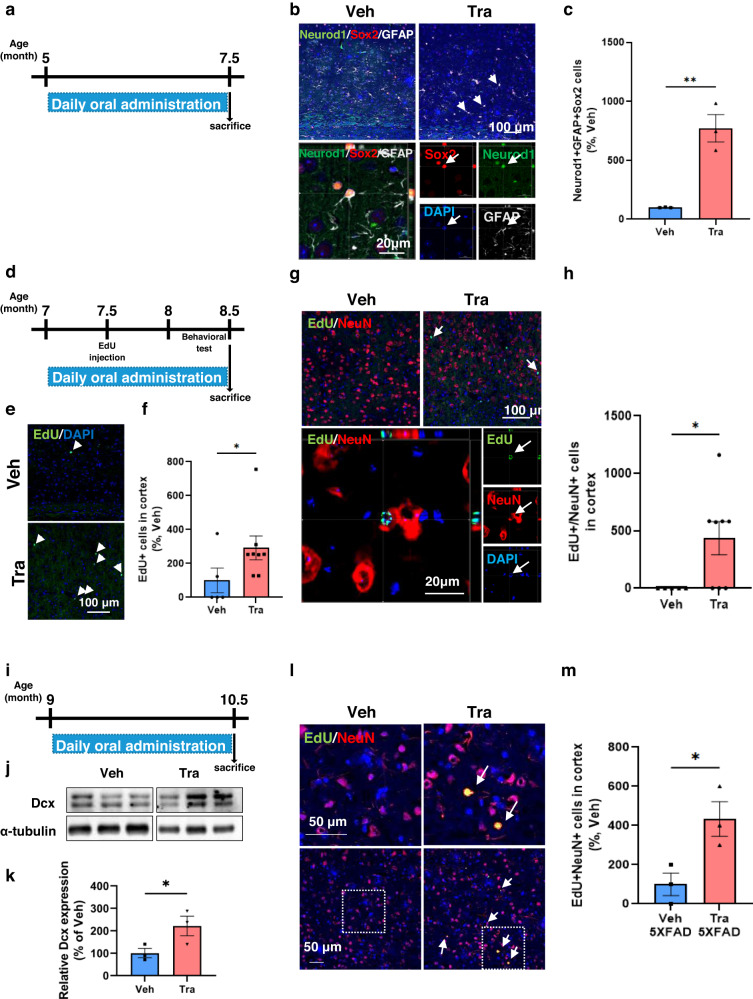
Trametinib induces adult cortical neurogenesis in the 5XFAD mouse brain. **a** Scheme of trametinib administration to 5XFAD mice for (**b**) and (**c**). Trametinib was administered to 5-month-old 5XFAD mice for 2.5 months. **b**, **c** Immunofluorescence staining images (**b**) and quantification (**c**) of Neurod1^+^ Sox2^+^ Gfap^+^ triple-positive cells in the cortex of 7.5-month-old 5XFAD mice. Arrows indicate triple-positive cells. **b** The lower panel represents triple-positive cells in a high-magnification image. *n* = 3 mice per group; sagittal sections from each mouse. Scale bars, 20 or 100 μm. Normalized to the 5XFAD-vehicle group. **d** Scheme of trametinib administration to 5XFAD mice for (**e**) and (**f**). Trametinib was administered to 7-month-old 5XFAD mice for 1.5 months. **e**, **f** Images for EdU incorporation analysis (**e**) and changes in EdU-positive cell numbers (**f**) in the cortex of 8.5-month-old 5XFAD mice. Arrows indicate stained cells. *n* = 3 mice per group, *n* = 2 ~ 3 serial sagittal sections from each mouse. Scale bars, 100 μm. Normalized to the 5XFAD-vehicle group. **g**, **h** Immunofluorescence staining images (**g**) and quantification of EdU+NeuN+ cell numbers (**h**) in the cortex of 8.5-month-old 5XFAD mice. Arrows indicate stained cells. The lower panel represents EdU+ NeuN+ double-positive cells at high magnification. *n* = 3 mice per group, *n* = 2 ~ 3 serial sagittal sections from each mouse. Scale bars, 20 or 100 μm. Normalized to the 5XFAD-Trametinib group. **i** Scheme of trametinib administration to 5XFAD mice for (**j**–**m**). Trametinib was administered to 9-month-old 5XFAD mice for 1.5 months. **j**, **k** Dcx level in the lysate of the cortex of 11-month-old 5XFAD mice (**j**) and quantification of band intensity (**k**). **l**, **m** Immunohistostaining (**l**) and counting (**m**) of NeuN+ EdU+ double-positive cells in the cortex of 11-month-old 5XFAD mice. Arrows indicate stained cells. *n* = 3 mice per group; sagittal sections from each mouse. Scale bars, 50 μm. Data are representative of three independent experiments, and values are expressed as the mean ± SEM. *P* values were obtained by Student’s *t*-test. *p < 0.05 and **p < 0.005.

We asked whether trametinib can induce 5XFAD mouse adult cortical neurogenesis, which is characterized by neuronal loss in brain cortical layer V^39^. Previous studies have demonstrated that the production of new neurons occurs in the cortex after cortical injury, and those new neurons originate from cortical glial cells or NSCs migrating from the SVZ^40–42^. Therefore, we investigated which cell population differentiated into cortical neurons. We counted the number of Sox2, GFAP, and Neurod1 triple-positive cells in sagittal sections of the 5XFAD mouse somatosensory cortex. Sox2 and GFAP double-positive cells are well-known as neural stem/progenitor cells in the DG and SVZ^43^. Sox2, in particular, is known as a reprogramming factor in the adult brain^44^, and Neurod1 is expressed in dividing neural progenitor cells^44^. Thus, these triple-positive (Neurod1+/Sox2+/GFAP+) cells may act as neurogenic progenitors in the cortex. After 0.1 mg/kg trametinib was administered to five-month-old 5XFAD mice for 2.5 months, mice were sacrificed at the age of 7.5 months (Fig. 3a). Interestingly, triple-positive cell numbers were significantly increased by trametinib administration in the 5XFAD mouse cortex (Fig. 3b, c). We also confirmed cortical neurogenesis using EdU incorporation analysis. We administered trametinib for 1.5 months to 7-month-old 5XFAD mice while giving them 200 mg/kg EdU injection 30 days before sacrifice at 8.5 months of age (Fig. 3d). The number of EdU-positive cells in the cortex increased with trametinib administration (Fig. 3e, f). Furthermore, the number of EdU/NeuN double-positive cells significantly increased (Fig. 3g, h), suggesting that trametinib distinctly induced neurogenesis and produced new neurons in the cortex. We also questioned whether trametinib can induce cortical neurogenesis in the late-symptomatic stage of AD and administered trametinib to 9-month-old 5XFAD mice for 1.5 months (Fig. 3i). The level of Dcx in the cortex was increased by trametinib administration (Fig. 3j, k), and EdU/NeuN double-positive cell numbers were also increased (Fig. 3l, m). These results strongly suggest that NPCs exist in the AD cortex and that trametinib activates cortical neurogenesis in 5XFAD mice.

To determine whether activation of neurogenesis by trametinib is a sufficient or necessary mechanism for recovery of cognitive impairment, we examined the effect of trametinib upon inhibition of neurogenesis using temozolomide (TMZ, neurogenesis inhibitor^45^) on 5XFAD mice. Compared with trametinib administration, trametinib and TMZ combined treatment did not rescue cognitive function (Supplementary Fig. 7a). In addition, hippocampal neurogenesis was not recovered, and the number of newborn neurons was not increased (Supplementary Fig. 7b, c). From these results, we recognize that activation of neurogenesis is important for recovery of cognitive function in 5XFAD mice.

### Trametinib induces functional rescue of AD pathogenesis by restoring neuron numbers and neuronal structure

**Fig. 4:**
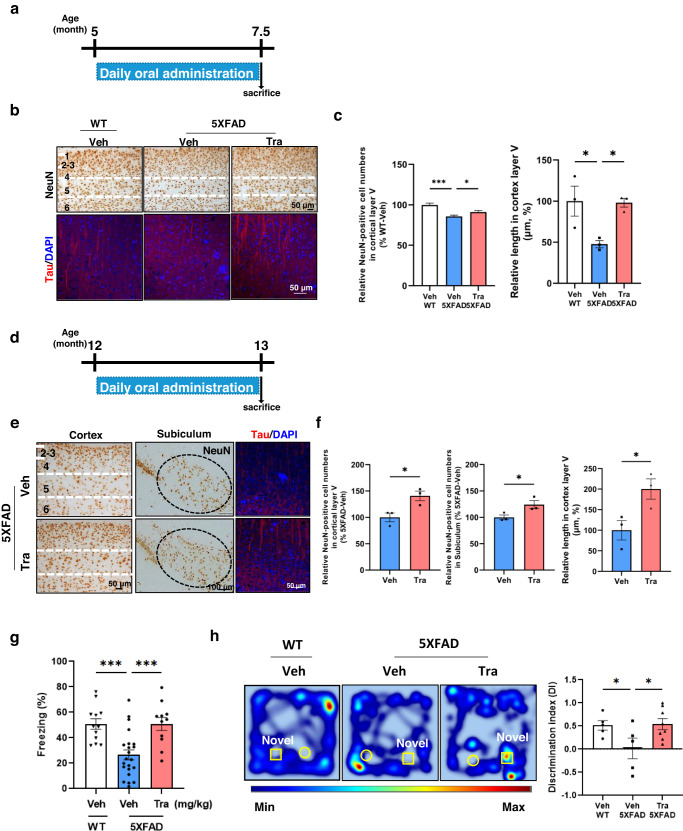
Trametinib induces neuroregeneration and recovers functional impairment. **a** Scheme of trametinib administration to 5XFAD mice for (**b**) and (**c**). Trametinib was administered to 5-month-old 5XFAD mice for 2.5 months. **b**, **c** Analysis of the cortex from 7.5-month-old 5XFAD mice. Sagittal sections of the cortex were immunostained for NeuN and Tau. The number of NeuN+ cells was counted in cortical layer V. *n* = 14 serial sagittal sections from each mouse, *n* = 3 mice per group. Normalized by cortical layer V area and renormalized to the WT-vehicle group. The length of tau-stained cells was measured in cortical layer V. *n* = 3 mice per group. *P* values were obtained by one-way ANOVA. **d** Scheme of trametinib administration experiments with 5XFAD mice for (**e**) and (**f**). Trametinib was administered to 12-month-old 5XFAD mice for 1 month. **e**, **f** Analysis of the cortex from 13-month-old 5XFAD mice. Sagittal sections of the cortex were immunostained for NeuN and Tau. The number of NeuN+ cells was counted in cortical layer V and subiculum. *n* = 21 serial sagittal sections from each mouse, *n* = 3 mice per group. Normalized by cortical layer V area and renormalized to the 5XFAD-vehicle group. Scale bars, 50 μm. The length of tau-stained cells was measured in cortical layer V. *n* = 3 mice per group. **g** Nine-month-old 5XFAD mice were administered vehicle or trametinib for 1.5 months. A fear conditioning test was performed, and the average freezing % in 2 min was calculated. *n* = 12 mice for the WT-vehicle group, *n* = 22 mice for the 5XFAD-vehicle group, *n* = 11 for the 5XFAD-trametinib group. *P* values were obtained by one-way ANOVA. **h** Seven-month-old 5XFAD mice were administered vehicle or trametinib for 1.5 months. A novel object recognition test was performed. Heatmap analysis of animal tracking following the novel object recognition test (left panel) and the discrimination index in 5 minutes was calculated (right panel). *n* = 5 mice for the WT-vehicle group, *n* = 5 mice for the 5XFAD-vehicle group, *n* = 8 for the 5XFAD-trametinib group. Data are representative of three independent experiments, and values are expressed as the mean ± SEM. *P* values were obtained by one-way ANOVA (**c**, **g**) and Student’s *t* test (**f**, **h**). *p < 0.05 and ***p < 0.001. Data quantification was performed blindly with respect to the experimental group.

To investigate whether adult neurogenesis, especially cortical neurogenesis, induced by trametinib supports the restoration of neurons in 5XFAD mice, we administered daily 0.1 mg/kg trametinib to 5-month-old 5XFAD mice for 2.5 months (Fig. 4a) or 12-month-old 5XFAD mice for 1 month (Fig. 4d). The number and axonal length of neurons in cortical layer V of the somatosensory cortex were decreased in the 7.5-month-old trametinib-treated 5XFAD mice, whereas 0.1 mg/kg trametinib administration for 2.5 months to 5-month-old 5XFAD mice improved neuronal numbers and axonal length (Fig. 4b, c). The number and axonal length of neurons in cortical layer V of the cortex and subiculum were also significantly increased in 13-month-old 5XFAD mice upon trametinib administration compared with vehicle-treated 5XFAD mice (Fig. 4e, f). This was an unexpected effect of trametinib because, at this late stage, neuronal loss in the cortex is believed to be too severe to allow restoration.

Consequently, we further examined whether trametinib administration recovers AD pathologies and improves cognitive functions in 5XFAD mice. Administration of 0.1 mg/kg trametinib for 1.5 months to 9-month-old 5XFAD mice decreased the size of amyloid plaques in the cortex (Supplementary Fig. 8). The neuronal network stability in the cortex was also evaluated by observing the epileptic discharges (Eds) evoked by 4-aminopyridine (4AP) and bicuculline treatment in slices extracted from the temporoparietal cortex via high-density microelectrode array (HD MEA) recording (Supplementary Fig. 9a). EDs reflect the spreading of the local field potential (LFP) from the origin of onset^46^. The number of effective activation electrodes in the recording slices was significantly reduced in the 5XFAD mice, which was recovered by oral trametinib treatment. In addition, to confirm the recovery of neuronal structures by trametinib, we checked the level of synaptophysin (presynaptic marker) in EdU+ cells. The intensity of synaptophysin was recovered by trametinib administration (Supplementary Fig. 9b). Furthermore, in the fear conditioning test and novel object recognition test, vehicle-administered 5XFAD mice showed cognitive impairment, whereas administration of 0.1 mg/kg trametinib for 1.5 months to 9-month-old 5XFAD mice or for 1.5 months to 7-month-old 5XFAD mice improved cognitive functions as shown by an increase in the percentage of freezing or discrimination index measures (Fig. 4g, h) with no changes in locomotor functions (Supplementary Fig. 10a, b). In addition, there was no significant difference in freezing rate between WT and 5XFAD mice before the fear conditioning test (Supplementary Fig. 10c). However, trametinib treatment did not result in a further increase in neurogenesis-related markers or cognitive function in WT mice (Supplementary Fig. 11). These data indicate that trametinib recovers damaged neurons and their networks and rescues cognitive function in 5XFAD AD model mice. Interestingly, trametinib can play a role in increasing the function of neurogenesis in the disrupted brain, but it may no longer improve cognitive function and neurogenesis activation in WT mice.

### Trametinib induces neurogenic differentiation of AD patient iPSC-derived NSCs

**Fig. 5:**
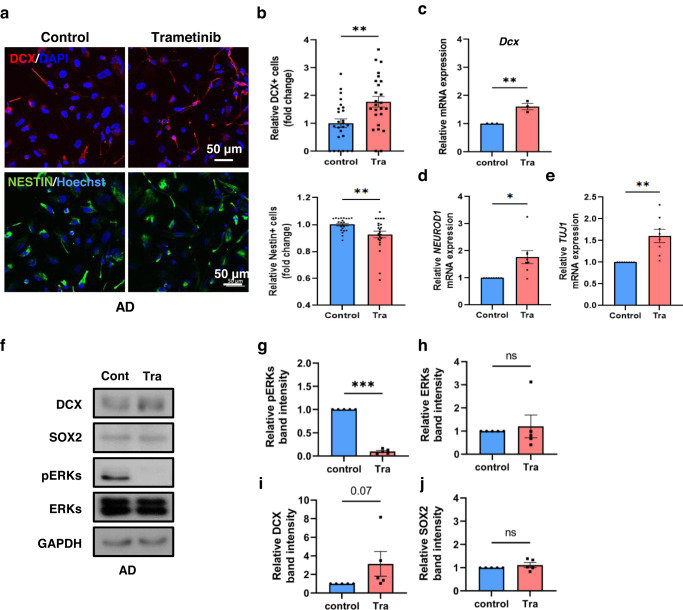
Neurogenic differentiation by trametinib in iPSC-derived NSCs from AD patients. AD patient iPSC-derived NSCs were cultured with 100 nM trametinib for 48 h. **a**, **b** Immunofluorescence staining of DCX and NESTIN (**a**) and quantification data for DCX+ cell or NESTIN+ cell numbers (**b**). Scale bars, 50 μm. **c**–**e** *DCX* (**c**), *NEUROD1* (**d**) or *TUJ1* (**e**) were detected using qRT‒PCR. Each sample was normalized to the expression of GAPDH. **f**–**j** Cell lysates were subjected to immunoblot analyses of DCX, SOX2, pERKs, ERKs and GAPDH (**f**) and quantified with band intensities. Normalized by GAPDH band intensity and renormalized to the control group (**g**–**j**). Statistical analysis was performed by three independent experiments. *P* values were obtained by Student’s *t*-test. *p < 0.05, **p < 0.005 and ***p < 0.001.

Next, we asked if the neurogenic effect of trametinib observed in 5XFAD AD model mice has human relevance using NSCs derived from human iPSCs. To assess the neurogenic effect of trametinib on AD patient iPSC-derived NSCs, cells were immunostained with DCX and NESTIN for 2 days after trametinib treatment. The number of NESTIN+ cells (NSC marker) was decreased, and DCX+ cells were increased by trametinib (Fig. 5a, b). The mRNA levels of the neurogenic markers *DCX, NEUROD1* and *TUJ1* were also increased in trametinib-treated NSCs (Fig. 5c–e). The level of pERKs was significantly decreased (Fig. 5f–h), while the level of the early postmitotic neuronal marker DCX was increased by trametinib (Fig. 5i, j). These neurogenic effects of trametinib were also observed in healthy donor iPSC-derived NSCs (Supplementary Fig. 12). From these data, we suggest that trametinib has the potential to induce neurogenic differentiation in AD patients.

## Discussion

AD cannot be explained by a single causal factor since it develops through complex interplay among neuronal cells throughout the brain^47,48^. Aβ aggregation, autophagic defects, and inflammatory responses form a negative feedback loop toward neuronal death and subsequently defective neural activity^49^. The development of AD therapeutics has focused on the elimination of toxic materials such as Aβ plaques or tau tangles. With the FDA’s recent approval of aducanumab, which targets the elimination of Aβ, controversy has arisen because the decision was made based on a surrogate endpoint—a significant reduction in Aβ plaques—rather than on a clinical endpoint^50^. Therefore, new strategies for the development of disease-modifying drugs are still needed.

Our study originated from the idea that adult neuronal differentiation from NSCs could be an effective therapeutic approach against various neurodegenerative diseases. In a previous study by Moreno-Jimenez et al. published in 2019^8^, adult hippocampal neurogenesis (AHN) was observed even up to the age of 90, but a decrease in Dcx+ cells was observed in AD patients. By confirming that the number gradually decreased according to disease progression, it was explained that disruption of AHN in the early stage of AD mediated AD pathogenesis and that disruption of AHN is an important part of AD. As an example, it has been reported that when AHN is genetically ablated in mice, neural loss is increased, and cognitive dysfunction is also induced^51^. In addition, when endogenous neurogenesis is augmented, memory impairments are restored in AD model mice^52^. Therefore, as a strategy to promote endogenous neurogenesis in AD, therapies that target the activation of neurogenesis, such as allopregnanolone and sovateltide, are being developed^53^. On the other hand, neurogenesis is restricted in the adult mammalian brain, but some studies have shown that astrocytes may be useful for neuronal replacement strategies after injury^54,55^. Therefore, we believe that activation of endogenous adult neurogenesis can sufficiently restore behavioral defects in 5XFAD mice.

We established a regenerative drug screening platform, ATRIVIEW^®^, using adult NSCs from Tg2576 mice, which produce an Aβ-induced toxic environment^23^. Of the screened compounds, trametinib was not only the most effective in inducing neuronal differentiation but also protective of the differentiated neuron-like cells in the Aβ_1–42_ oligomer-induced toxic environment. In particular, neuronal differentiation was accompanied by cell cycle arrest and proneuronal factor expression via trametinib. Moreover, we provide evidence that trametinib manifests its potential as an inducer of neuronal differentiation in AD patient iPSC-derived NSCs as well as adult NSCs derived from 5XFAD AD model mice.

In 5XFAD mice, we demonstrated that oral administration of trametinib restores impaired neurogenesis in the DG and SVZ. The 5XFAD mice express human amyloid precursor protein (APP) and presenilin1 (PSEN1) with a total of five AD-linked mutations^39^. These mice have a relatively early and aggressive presentation of amyloid plaques compared with other AD model mice. Furthermore, neuronal loss is also evident in the 5XFAD mice in the cortex and hippocampal subiculum. In the DG of 5XFAD mice, NSCs did not efficiently differentiate into neuroblasts or mature neurons (Fig. 2b, c). In the SVZ of 5XFAD mice, the differentiation of NSCs into neuroblasts (type A cells) was severely impaired (Fig. 2d, e). Both this process and the transition of type B1 NSCs to type C cells are induced by trametinib administration. In healthy brains, neuroblasts generated in the SVZ migrate to the OB via the RMS (rostral migratory stream), which is not observed in 5XFAD mice. Trametinib successfully replenished neuroblasts in the OB, which migrated from the SVZ (Fig. 2d). However, in WT mice, no further increase in cognitive function or changes in neurogenesis-related markers were observed with trametinib administration (Supplementary Fig. 11)^21^. When trametinib is administered to 5XFAD mice, it can play a role in increasing the function of the neurogenesis/autophagy-lysosome pathway (ALP) to the WT level; however, in the case of WT, neurogenesis or ALP is not disrupted, and ERK is not hyperactivated, so we think SNR1611 no longer improves cognitive function. In particular, by confirming that inhibition of neurogenesis in 5XFAD mice blocked the recovery of cognitive function induced by trametinib, neurogenesis appears to be essential for cognitive recovery from a neurogenesis-compromised state. In addition, there are several pieces of evidence for cortical neurogenesis in the 5XFAD mouse brain. We found that NPCs exist in the 5XFAD mouse brain cortex through immunohistochemical analyses (Fig. 3). Newborn neurons detected by EdU/NeuN double staining were also increased in the cortex by trametinib administration. Thus, it is possible that the activation of cortical neurogenesis by trametinib supports the increase in neuron number in the cortex of 5XFAD mice and the rescue of cognitive impairment.

Finally, we examined the possibility of clinical translation of trametinib using AD iPSC-derived NSCs. We used familial AD (PSEN1 M146I) iPSC-derived NSCs and confirmed that trametinib could induce neuronal differentiation. The PSEN1 M146I mutation is a widespread mutation in AD patients with PSEN1 mutations^56^. Therefore, it is reasonable to expect that trametinib can induce neurogenesis in an AD patient’s brain.

It is known that adult neurogenesis is disrupted in AD patients^57^. Several studies have shown that transit amplifying cells, involved in the early stages of neurogenesis in the hippocampus, are increased in a pathogenic environment, but their maturation is defective and unable to proceed further^8^. Our results suggest that restoring endogenous neurogenesis in the DG/SVZ and the cortex can contribute to recovery from AD.

Notably, our research showed that trametinib restores the supply of newly generated neurons even in the severe stage of the disease, at least in the AD animal model. In addition, trametinib induces neurogenic differentiation of AD patient iPSC-derived NSCs. These findings indicate that the regenerative approach using trametinib would be effective in treating AD and other neurodegenerative diseases.

### Supplementary information

Supplementary information

## References

[1] Murphy KE, Park JJ (2017). Can co-activation of Nrf2 and neurotrophic signaling pathway slow Alzheimer’s disease?. Int J. Mol. Sci..

[2] Sala Frigerio C, De Strooper B (2016). Alzheimer’s disease mechanisms and emerging roads to novel therapeutics. Annu. Rev. Neurosci..

[3] Fleifel D (2018). Recent advances in stem cells therapy: a focus on cancer, Parkinson’s and Alzheimer’s. J. Genet. Eng. Biotechnol..

[4] De Gioia R (2020). Neural stem cell transplantation for neurodegenerative diseases. Int. J. Mol. Sci..

[5] Gould E (2007). How widespread is adult neurogenesis in mammals?. Nat. Rev. Neurosci..

[6] Magnusson JP, Frisen J (2016). Stars from the darkest night: unlocking the neurogenic potential of astrocytes in different brain regions. Development.

[7] Kozareva DA (2019). Born this way: Hippocampal neurogenesis across the lifespan. Aging Cell.

[8] Moreno-Jimenez EP (2019). Adult hippocampal neurogenesis is abundant in neurologically healthy subjects and drops sharply in patients with Alzheimer’s disease. Nat. Med..

[9] Perry EK (2012). Neurogenic abnormalities in Alzheimer’s disease differ between stages of neurogenesis and are partly related to cholinergic pathology. Neurobiol. Dis..

[10] Li J (2007). MEK/ERK signaling contributes to the maintenance of human embryonic stem cell self-renewal. Differ.; Res. Biol. Diversity.

[11] Chambard JC (2007). ERK implication in cell cycle regulation. Biochim. Biophys. Acta.

[12] McCubrey JA (2007). Roles of the Raf/MEK/ERK pathway in cell growth, malignant transformation and drug resistance. Biochim. Biophys. Acta.

[13] Callihan P (2014). Convergent regulation of neuronal differentiation and Erk and Akt kinases in human neural progenitor cells by lysophosphatidic acid, sphingosine 1-phosphate, and LIF: specific roles for the LPA1 receptor. ASN Neuro.

[14] Phoenix TN, Temple S (2010). Spred1, a negative regulator of Ras-MAPK-ERK, is enriched in CNS germinal zones, dampens NSC proliferation, and maintains ventricular zone structure. Genes Dev..

[15] Wang Y (2012). ERK inhibition rescues defects in fate specification of Nf1-deficient neural progenitors and brain abnormalities. Cell.

[16] Moon BS (2011). Sur8/Shoc2 involves both inhibition of differentiation and maintenance of self-renewal of neural progenitor cells via modulation of extracellular signal-regulated kinase signaling. Stem Cells.

[17] Sabelstrom H (2019). Driving neuronal differentiation through reversal of an ERK1/2-miR-124-SOX9 axis abrogates glioblastoma aggressiveness. Cell Rep..

[18] Wang Z (2019). Amyloid-beta1-42 dynamically regulates the migration of neural stem/progenitor cells via MAPK-ERK pathway. Chem. Biol. Interact..

[19] Legrier ME (2001). Region-specific expression of cell cycle inhibitors in the adult brain. Neuroreport.

[20] Robledinos-Anton N (2020). TAZ represses the neuronal commitment of neural stem cells. Cells.

[21] Chun YS (2022). MEK1/2 inhibition rescues neurodegeneration by TFEB-mediated activation of autophagic lysosomal function in a model of Alzheimer’s Disease. Mol. Psychiatry.

[22] Kim MY (2013). Isolation and maintenance of cortical neural progenitor cells in vitro. Methods Mol. Biol..

[23] Baldassarro VA (2013). Neural stem cells isolated from amyloid precursor protein-mutated mice for drug discovery. World J. Stem Cells.

[24] Meyer K (2019). REST and neural gene network dysregulation in iPSC models of Alzheimer’s disease. Cell Rep..

[25] von Bohlen Und Halbach O (2007). Immunohistological markers for staging neurogenesis in adult hippocampus. Cell Tissue Res..

[26] Konirova J (2017). Modulated DISP3/PTCHD2 expression influences neural stem cell fate decisions. Sci. Rep..

[27] Ansorg A (2015). Immunohistochemistry and multiple labeling with antibodies from the same host species to study adult hippocampal neurogenesis. J. Vis. Exp..

[28] Sollner JF (2017). An RNA-Seq atlas of gene expression in mouse and rat normal tissues. Sci. Data.

[29] Singh A (2015). Targeted inhibition of MEK1 by cobimetinib leads to differentiation and apoptosis in neuroblastoma cells. J. Exp. Clin. Cancer Res..

[30] Saqub H (2020). Dinaciclib, a cyclin-dependent kinase inhibitor, suppresses cholangiocarcinoma growth by targeting CDK2/5/9. Sci. Rep..

[31] Fleisher AS (2008). Phase 2 safety trial targeting amyloid beta production with a gamma-secretase inhibitor in Alzheimer disease. Arch. Neurol..

[32] Raskin J (2007). Efficacy of duloxetine on cognition, depression, and pain in elderly patients with major depressive disorder: an 8-week, double-blind, placebo-controlled trial. Am. J. Psychiatry.

[33] Song H (2016). Stress-induced nuclear translocation of CDK5 suppresses neuronal death by downregulating ERK activation via VRK3 phosphorylation. Sci. Rep..

[34] Pei JJ (2002). Up-regulation of mitogen-activated protein kinases ERK1/2 and MEK1/2 is associated with the progression of neurofibrillary degeneration in Alzheimer’s disease. Brain Res Mol. Brain Res..

[35] Kirouac L (2017). Activation of Ras-ERK signaling and GSK-3 by amyloid precursor protein and amyloid beta facilitates neurodegeneration in Alzheimer’s disease. eNeuro.

[36] Yamaguchi T (2007). Identification of JTP-70902, a p15(INK4b)-inductive compound, as a novel MEK1/2 inhibitor. Cancer Sci..

[37] Goldman S (2003). Glia as neural progenitor cells. Trends Neurosci..

[38] Gomez HG (2019). Suppressor of Fused regulates the proliferation of postnatal neural stem and precursor cells via a Gli3-dependent mechanism. Biol. Open.

[39] Oakley H (2006). Intraneuronal beta-amyloid aggregates, neurodegeneration, and neuron loss in transgenic mice with five familial Alzheimer’s disease mutations: potential factors in amyloid plaque formation. J. Neurosci..

[40] Faiz M (2015). Adult neural stem cells from the subventricular zone give rise to reactive astrocytes in the cortex after stroke. Cell Stem Cell.

[41] Saha B (2013). Cortical lesion stimulates adult subventricular zone neural progenitor cell proliferation and migration to the site of injury. Stem Cell Res..

[42] Vargas-Saturno L, Ayala-Grosso C (2018). Adaptive neurogenesis in the cerebral cortex and contralateral subventricular zone induced by unilateral cortical devascularization: possible modulation by dopamine neurotransmission. Eur. J. Neurosci..

[43] Ming GL, Song H (2011). Adult neurogenesis in the mammalian brain: significant answers and significant questions. Neuron.

[44] Gao Z (2009). Neurod1 is essential for the survival and maturation of adult-born neurons. Nat. Neurosci..

[45] Garthe A (2009). Adult-generated hippocampal neurons allow the flexible use of spatially precise learning strategies. PLoS ONE.

[46] Ferrea E (2012). Large-scale, high-resolution electrophysiological imaging of field potentials in brain slices with microelectronic multielectrode arrays. Front. Neural Circuits.

[47] Heppner FL (2015). Immune attack: the role of inflammation in Alzheimer disease. Nat. Rev. Neurosci..

[48] Wang J (2017). A systemic view of Alzheimer disease - insights from amyloid-beta metabolism beyond the brain. Nat. Rev. Neurol..

[49] Herrup K (2010). Reimagining Alzheimer’s disease–an age-based hypothesis. J. Neurosci..

[50] Knopman DS, Perlmutter JS (2021). Prescribing aducanumab in the face of meager efficacy and real risks. Neurology.

[51] Hollands C (2017). Depletion of adult neurogenesis exacerbates cognitive deficits in Alzheimer’s disease by compromising hippocampal inhibition. Mol. Neurodegener..

[52] Mishra R (2022). Augmenting neurogenesis rescues memory impairments in Alzheimer’s disease by restoring the memory-storing neurons. J. Exp. Med..

[53] Zhang Q (2023). Promoting endogenous neurogenesis as a treatment for Alzheimer’s disease. Mol. Neurobiol..

[54] Magnusson JP (2014). A latent neurogenic program in astrocytes regulated by Notch signaling in the mouse. Science.

[55] Zamboni M (2020). A widespread neurogenic potential of neocortical astrocytes is induced by injury. Cell Stem Cell.

[56] Lin YS (2020). Mutational analysis in familial Alzheimer’s disease of Han Chinese in Taiwan with a predominant mutation PSEN1 p.Met146Ile. Sci. Rep..

[57] Jin K (2004). Increased hippocampal neurogenesis in Alzheimer’s disease. Proc. Natl Acad. Sci. USA.

