# Supplementary material for: Trametinib activates endogenous neurogenesis and recovers neuropathology in a model of Alzheimer’s disease
Source: Exp Mol Med. 2023 Oct 2;55(10):2177–89. doi: 10.1038/s12276-023-01073-2 (PMC10618442; doi:10.1038/s12276-023-01073-2)
Supplement: Supplementary file 1 — Supplementary information [file 12276_2023_1073_MOESM1_ESM.pdf]

Supplementary Information for

Trametinib activates endogenous neurogenesis and recovers neuropathology in a model of Alzheimer's disease

Mi-Yeon Kim[1], Mi Jeong Kim[1], Changyeob Lee[3], Juwon Lee[3], Sang Seong Kim[4,8], Sungho Hong[5], Hyoung Tae Kim[1,9], Jinsoo Seo[3], Ki-Jun Yoon[6,7*], Sungho Han[1,2*]

[1]Neuroscience Research Center, Genuv Inc., Seoul 03175, Republic of Korea

[2]Head Office, Genuv Inc., Seoul 04520, Republic of Korea

[3]Department of Brain Sciences, Daegu Gyeongbuk Institute of Science and Technology (DGIST), Daegu 42988, Republic of Korea

[4]College of Pharmacy, Hanyang University ERICA, Gyeonggi-do 15588, Republic of Korea

[5]Computational Neuroscience Unit, Okinawa Institute of Science and Technology, Okinawa 904-0495, Japan

[6]Department of Biological Sciences, Korea Advanced Institute of Science and Technology (KAIST), Daejeon 34141, Republic of Korea

[7]KAIST Stem Cell Center, KAIST, Daejeon 34141, Republic of Korea

[8]Present address: Department of Biomedical Science and Engineering, Gwangju Institute of Science and Technology, Gwangju 61005, Republic of Korea

[9]Present address: Shaperon Inc., Seoul 06373, Republic of Korea

[*]Correspondence: han@genuv.com, kijunyoon@kaist.ac.kr

This PDF file includes:

Supplementary Fig. 1 to 12

Supplementary Table 1 to 4

Supplementary Figures

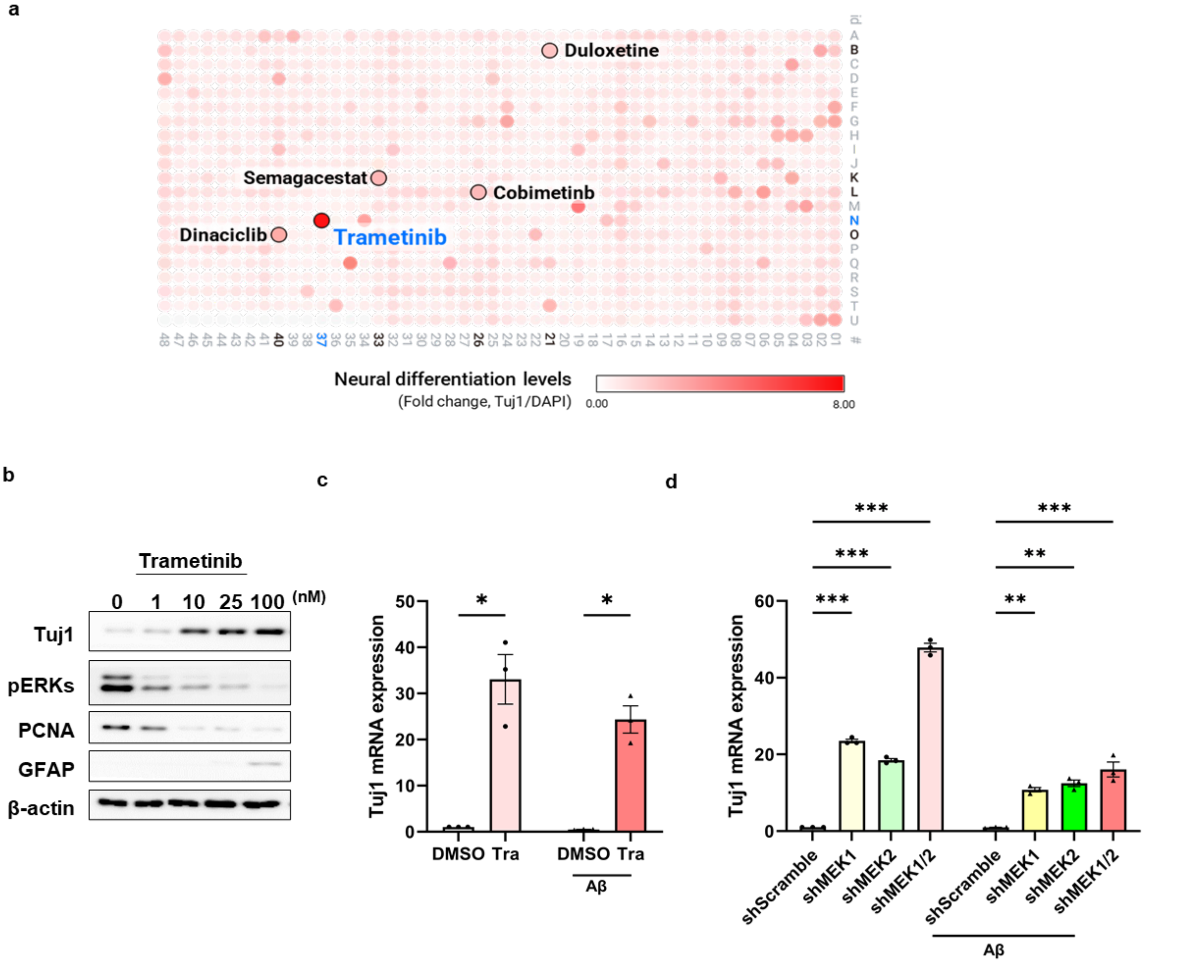

Supplementary Fig. 1. Inhibition of MEK/ERK signaling induces neuronal differentiation of adult NSCs. a Adult NSCs from Tg2576 mice were treated with FDA-approved drugs (994 compounds) at a final concentration of 0.5 μM. After 48 hrs, the level of Tuj1 was measured by immunocytochemistry. **b** Adult NSCs of C57BL/6 mice were cultured in the medium containing 20 ng/ml EGF and 20 ng/ml bFGF. Adult NSCs were treated with trametinib at indicated concentrations for 48 hrs. Cell lysates were subjected to immunoblot analyses. **c** Adult NSCs were treated with 100 nM of trametinib and 10 μM of A$\beta_{1\text{-}42}$ oligomers simultaneously for 48 hrs. *Tuj1* mRNA expression was analyzed using qRT-PCR and normalized to the expression of *Gapdh*. **d** Adult NSCs were transfected with shRNA against

MEK1 (shMEK1) and/or MEK2 (shMEK2) and further cultured for 48 hrs. The level of *Tuj1* mRNA was analyzed using qRT-PCR. Each sample was normalized to the expression of *Gapdh*. Data are representative of three independent experiments and values are expressed in mean ± SEM. Tra, trametinib treatment; Aβ, 10 μM of $A\beta_{1-42}$ oligomer treatment; Aβ+Tra, 10 μM of $A\beta_{1-42}$ oligomer and trametinib co-treatment. Statistical analysis was performed by 2-way ANOVA followed by Bonferroni analysis. *$p < 0.05$, **$p < 0.005$, ***$p < 0.001$.

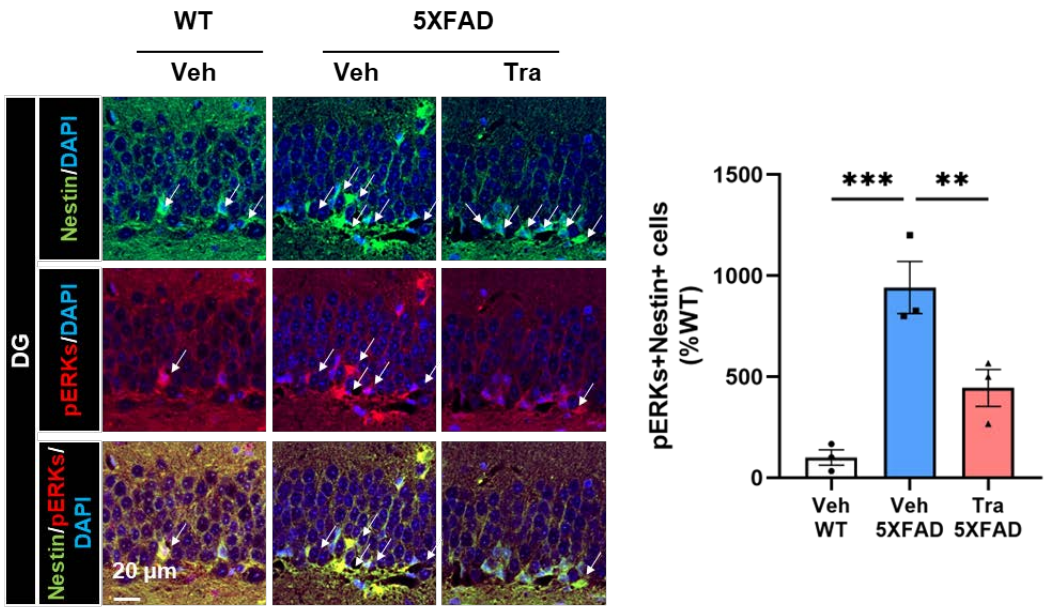

Supplementary Fig.2. Trametinib reduces hyperactivated pERKs in NSCs in the dentate gyrus of 5XFAD mouse brain. Trametinib was administered to 5-month-old 5XFAD mice for 2.5months. Immunofluorescence staining images and quantification of stained cell numbers in the hippocampal DG of 7.5-month-old 5XFAD mice. Arrows indicate Nestin and pERKs co-stained cells. n=3 mice per group; sagittal sections from each mouse brain. Scale bars, 20 µm. Normalized by the WT-vehicle group. Data are representative of three independent experiments and values are expressed in mean ± SEM. P values were obtained by one-way ANOVA. **$p < 0.005$ and ***$p < 0.001$.

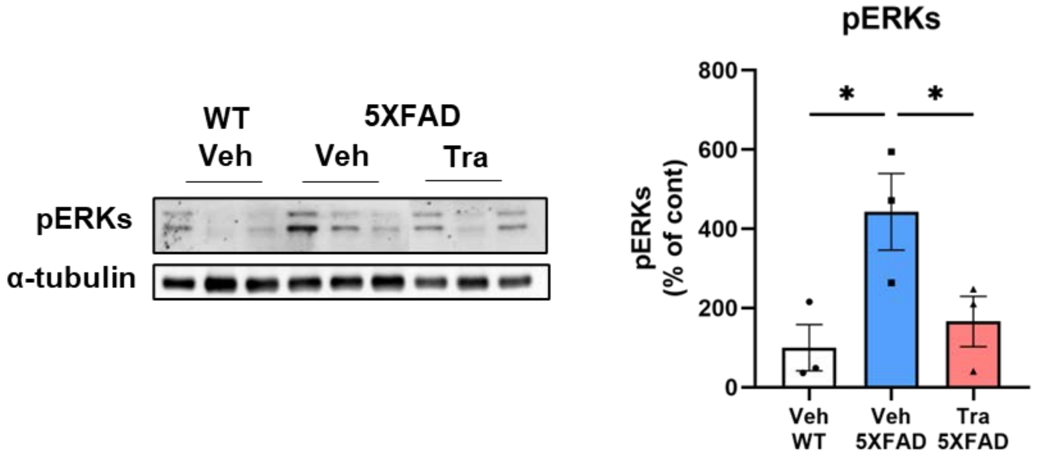

Supplementary Fig. 3. Trametinib reduces the level of pERKs in the 5XFAD brain. Trametinib was administered to 5-month-old 5XFAD mice for 2.5 months. Brain cortex lysates were subjected to immunoblot analyses of pERKs and α-tubulin and band intensities were quantified.

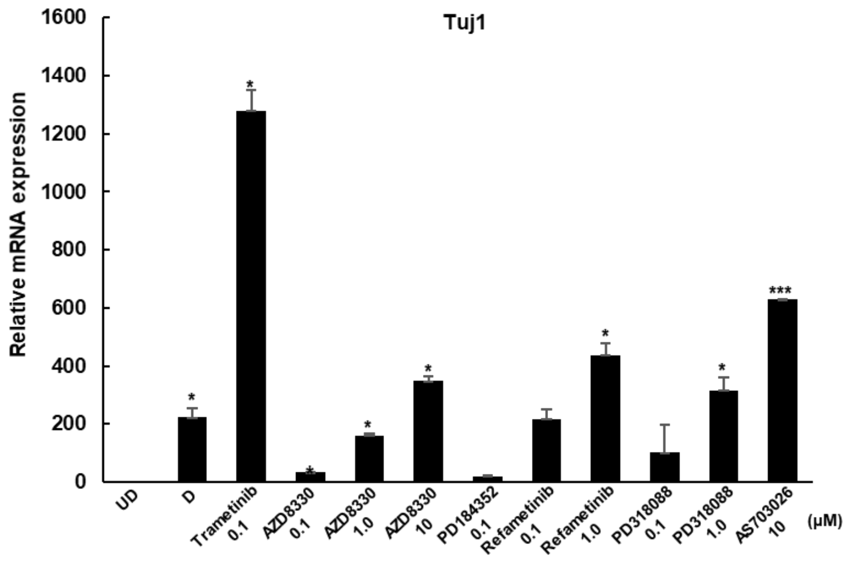

Supplementary Fig. 4. Inhibition of MEK/ERK signaling by MEK1/2 inhibitors induce neuronal differentiation of embryonic NSCs. Embryonic NSCs from E14.5 mice were treated with MEK1/2 inhibitors. After 48 hrs, the mRNA level of *Tuj1* was measured by qRT-PCR and normalized to the expression of *Gapdh*.

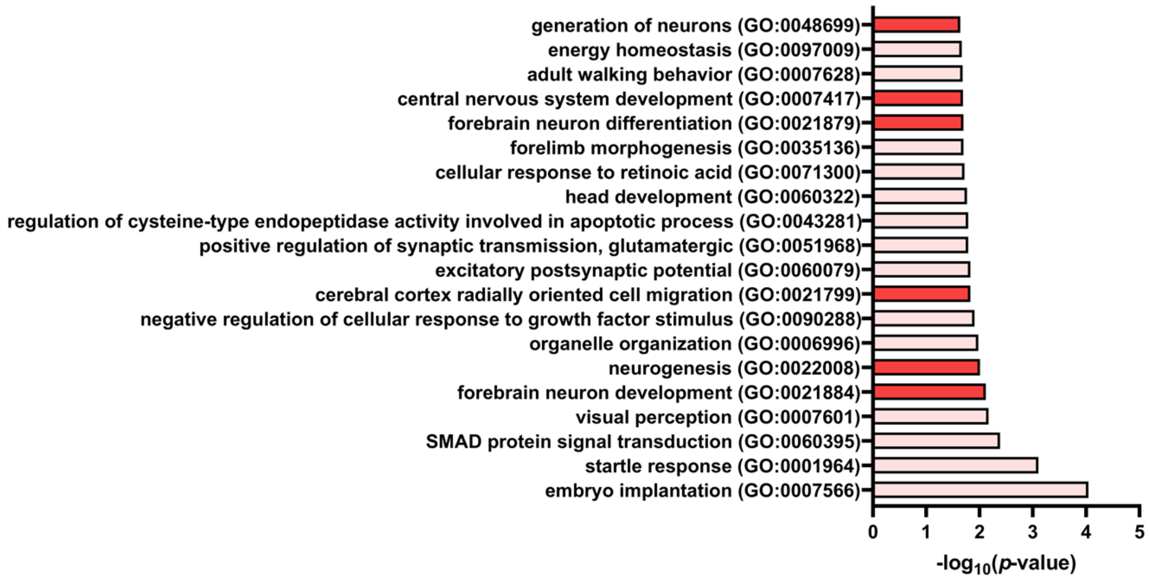

Supplementary Fig. 5. Transcriptomic analysis reveals that an abundance of upregulated genes by trametinib administration in normal mice are related with neurogenesis. GO term enrichment analysis on biological processes in the whole brain of normal mice after 2 weeks of trametinib treatment (FDR < 0.05).

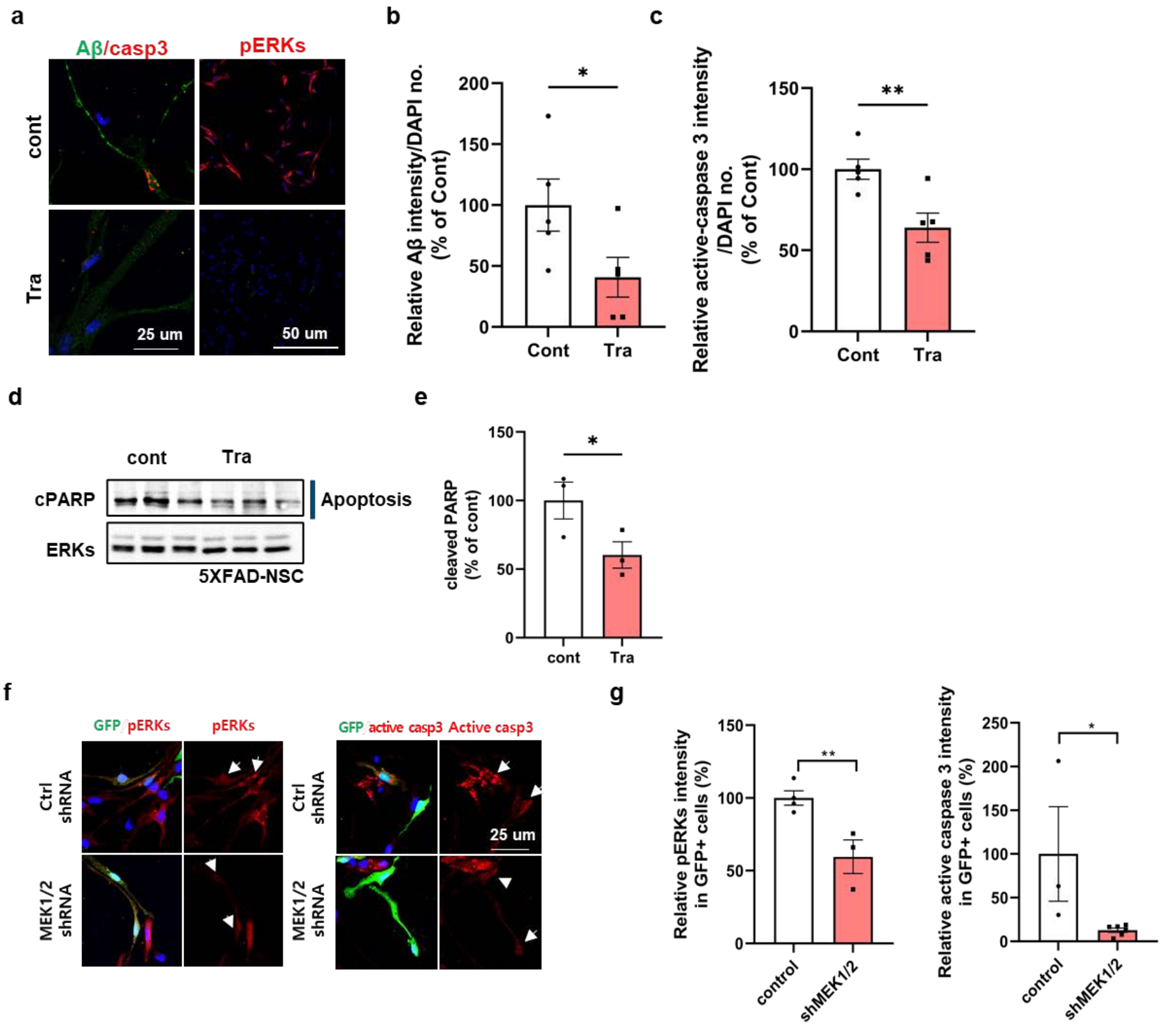

Supplementary Fig. 6. Stemness of NSCs from 5XFAD mice has defects, and trametinib reduces Aβ accumulation and apoptosis by inhibiting MEK/ERK signaling. a-e Adult NSCs from 5XFAD mice were seeded on plates and treated with 100 nM of trametinib in medium without growth factors for 48 hrs. **a-c** Immunofluorescence staining of active caspase 3 and Aβ, or phosphorylated ERK (**a**) and quantification of Aβ and active caspase 3 intensities (**b, c**). Scale bars, 25 μm or 50 μm. (**d, e**) Cell lysates were subjected to immunoblot analyses of cleaved PARP (**d**) and band intensities were quantified (**e**). **f, g** Adult NSCs from 5XFAD mice were transfected with shRNA-MEK1 and shRNA-MEK2 and further cultured for 48 hrs. Immunofluorescence staining of pERKs or active caspase 3 (**f**). Scale bars, 25 μm.

Quantification of pERKs and active caspase 3 intensities (**g**). Data are representative of three independent experiments and values are expressed in mean ± SEM. Tra, trametinib treatment; Aβ, 10 μM of $A\beta_{1\text{-}42}$ oligomer treatment; Aβ+Tra, 10 μM of $A\beta_{1\text{-}42}$ oligomer and trametinib co-treatment. Statistical analysis was performed by 2-way ANOVA followed by Bonferroni analysis. *$p < 0.05$, **$p < 0.005$, ***$p < 0.001$.

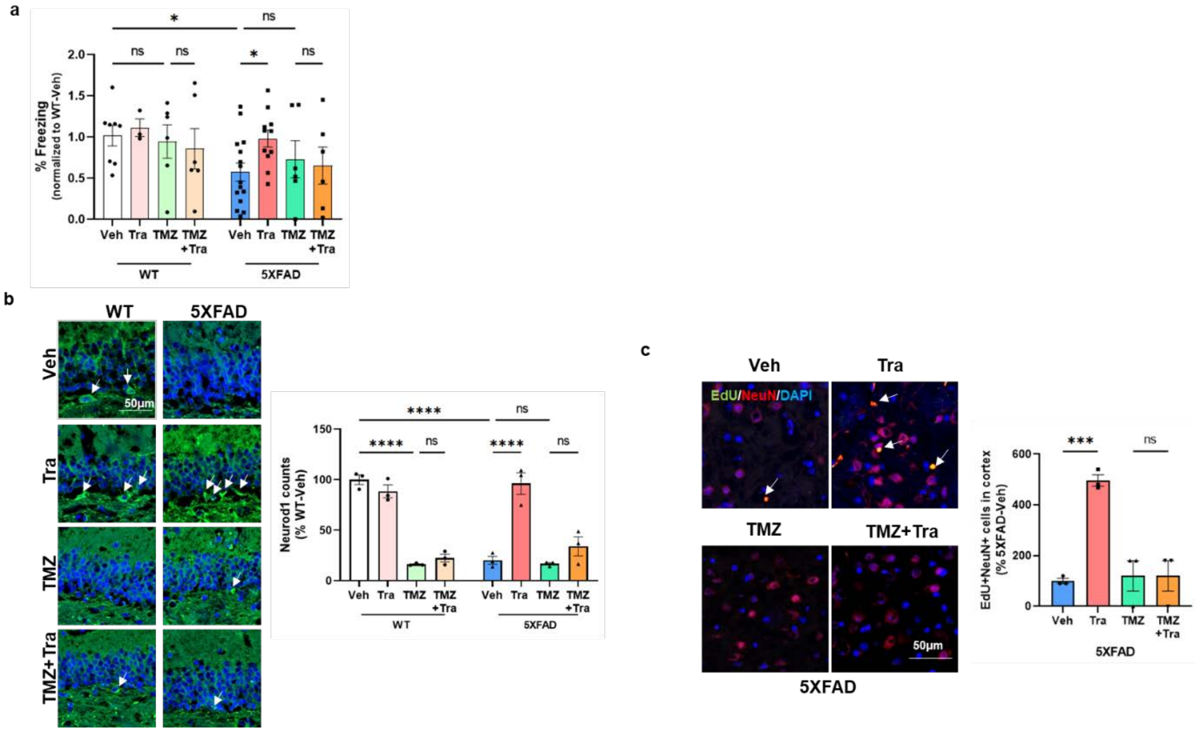

Supplementary Fig. 7. Activation of neurogenesis in 5XFAD mice is necessary for cognitive function. a Seven-month-old 5XFAD mice were administered with the vehicle or trametinib for 1.5 months. Fear conditioning test was performed, and the average of freezing % in 2 minutes was calculated. The freezing % was normalized by the WT-vehicle group. n=8 mice for WT-vehicle group, n=4 mice for WT-trametinib group, n=6 mice for WT-temozolomide (TMZ), n=6 mice for WT-TMZ+trametinib group, n=15 mice for 5XFAD-vehicle group, n=11 mice for 5XFAD-trametnib group, n=6 mice for 5XFAD-TMZ group, n=6 mice for 5XFAD-TMZ+trametinib group. P values were obtained by two-way ANOVA test. **b, c** Immunofluorescence staining of Neurod1 in hippocampal dentate gyrus (**b**) and EdU/NeuN in the cortex (**c**). Arrows indicate stained cells. n=3 mice per group; sagittal sections from each mouse. Scale bars, 50 μm. Data are representative of three independent experiments and values are expressed in mean ± SEM. P values were obtained by two-way ANOVA. *p < 0.05, **p < 0.005 and ***p<0.001.

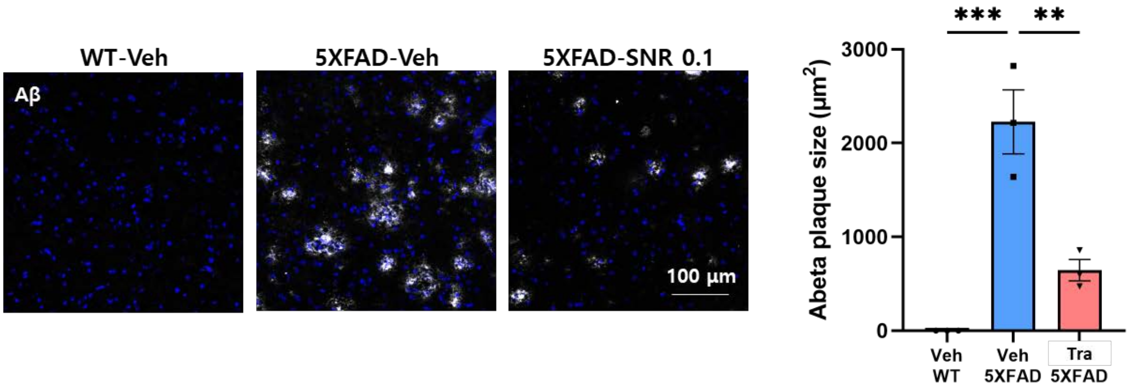

Supplementary Fig. 8. Trametinib reduces amyloid plaques in 5XFAD mice brain cortex. Trametinib was administered to 9-month-old 5XFAD mice for 1.5 months. Sagittal sections of the cortex were immunostained for Aβ. The area of amyloid plaques was measured. n=3 sagittal sections from each mouse, n=3 mice per group. Normalized to WT-vehicle group. Scale bars, 100 μm. P values were obtained by one-way ANOVA test. $^{**}p < 0.005$ and $^{***}p < 0.001$. Quantification of data was done blind with respect to the experimental groups.

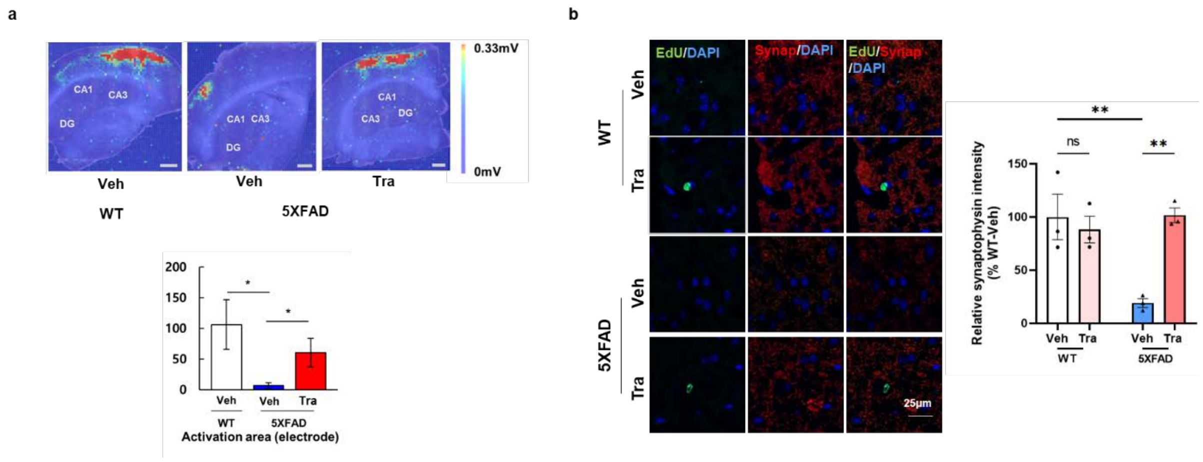

Supplementary Fig. 9. Trametinib rescues disrupted neuronal networks. a 0.1 mg/kg of trametinib was administered to 5-month-old 5XFAD mice for 2.5 months. MEA (Multi electrode assay) recording in cortical slices. Superimposition of the largest activation area during 4AP with bicuculline treatment in MEA over each slice image. Scale bars: 500 μm. Activation area represented by effective activation electrodes in the three groups. (n=16 slices in WT 5 mice, n=8 in 5XFAD-vehicle 3 mice, n=10 in 5XFAD-trametinib 3 mice). *P* values were obtained by one-way ANOVA test. *p < 0.05. **b** 0.1 mg/kg of trametinib was administered to 7-moth-old 5XFAD mice for 1.5 months. 200 mg/kg of EdU was also injected 30 days before sacrifice. Immunofluorescence staining images and quantification of synaptophysin intensity. n=3 sagittal sections from each mouse, n=3 mice per group. Normalized to WT-vehicle group. Scale bars, 25 μm. *P* values were obtained by two-way ANOVA test. **p < 0.005.

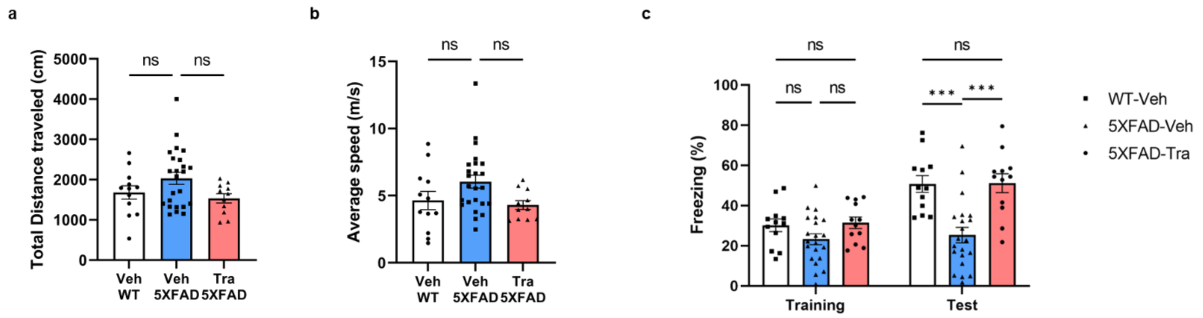

Supplementary Fig. 10. Motor function or basal freezing behavior of 5XFAD was not altered by vehicle or trametinib administration. a, b Nine-month-old 5XFAD mice were daily administered with vehicle or trametinib. Open field test was performed 29 days after the first administration. **a** Total traveled distance of vehicle- or trametinib-administered 5XFAD mice was measured for 10 minutes. **b** Average speed of vehicle- or trametinib-5XFAD mice was measured for 10 minutes. **c** The average of freezing % in 2 minutes was calculated during training (without electric shock) and during the test (with electric shock). n=12 mice for WT-vehicle group, n=22 mice for 5XFAD-vehicle group, n=11 for 5XFAD-trametinib group. *P* values were obtained by one-way ANOVA test.

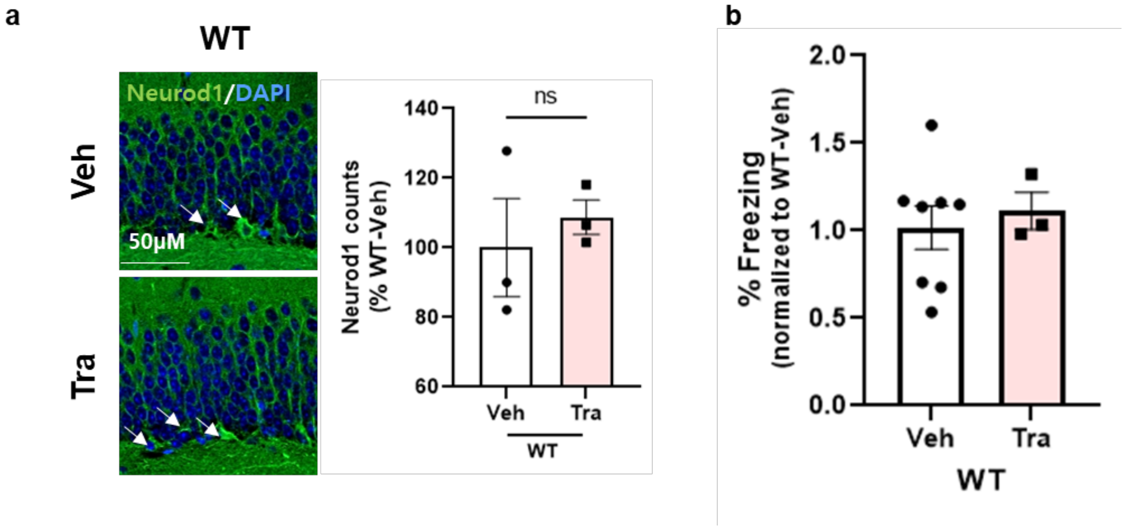

Supplementary Fig. 11. Neurogenesis or cognitive function of WT mice was not altered by trametinib administration. a, b Trametinib was administered to 7-month-old WT mice for 1.5 months. Immunofluorescence staining images and quantification of Neurod1-stained cell numbers (a) in the hippocampal DG. Arrows indicate stained cells. n=3 mice per group; sagittal sections from each mouse brain. Scale bars, 50 µm. Normalized by the WT-vehicle group. b Fear conditioning test was performed, and the average of freezing % in 2 minutes was calculated. The freezing % was normalized by the WT-vehicle group. n=8 mice for WT-vehicle group, n=3 mice for WT-trametinib group. Data are representative of three independent experiments and values are expressed in mean ± SEM. P values were obtained by Student's t-test. Quantification of data was done blind with respect to the experimental groups.

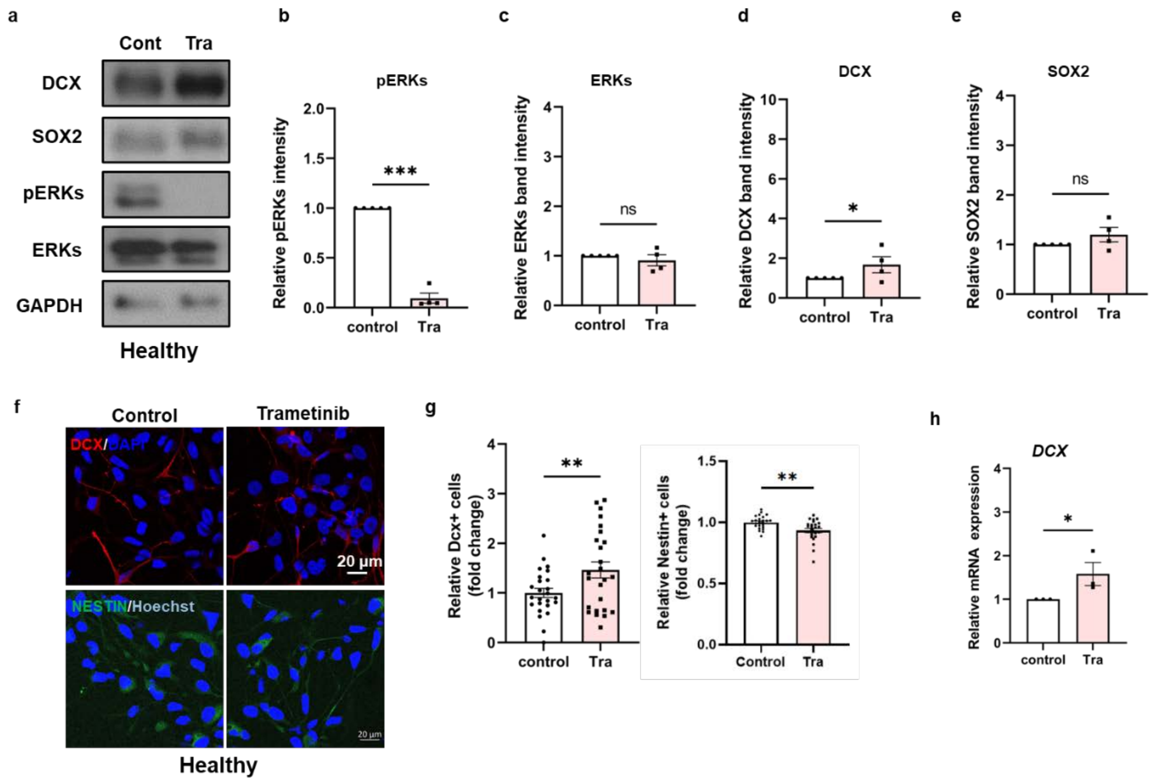

Supplementary Fig. 12. Neurogenic differentiation by trametinib in healthy donor-iPSC-derived NPCs. Healthy donor-iPSC-derived NPCs were cultured with 100 nM of trametinib for 48 hrs. **a-e** Cell lysates were subjected to immunoblot analyses of DCX, SOX2, pERKs, ERKs and GAPDH (**a**) and band intensities were quantified (**b-e**). **f, g** Immunofluorescence staining of DCX or NESTIN (**f**) and quantification data for DCX+ or NESTIN+ cell numbers (**g**). Scale bars, 20 μm. **h** *DCX* was detected using qRT-PCR. Each sample was normalized to the expression of *GAPDH*. Statistical analysis was done by three independent experiments. *P* values were obtained by Student's t-test. *p < 0.05, **p < 0.005 and ***p < 0.001.

Supplementary Table 1. Lists of upregulated Ngn2 target genes by trametinib

Gene_Symbol	Fold change
Ebf1	1.533571
Nhlh2	1.538540
Irx5	1.849281
Ebf3	1.900264
Irx3	1.992277
Sox14	2.132552
Cdh1	2.430865
Tead4	2.594590

Supplementary Table 2. List of antibodies

Antibody	Vendor	Cat. no.	Dilution for immunocyto-/ histochemistry	Dilution for western blot
Anti-Tuj1 mouse IgG	Cell signaling	4466	1:200	1:1000
Anti-pERKs rabbit IgG	Cell signaling	4370	1:200	1:1000
Anti-pERKs rabbit IgG	Cell signaling	9101		1:1000
Anti-ERKs rabbit IgG	Cell signaling	4695		1:1000
Anti-PCNA mouse IgG	Cell signaling	2586	-	1:1000
Anti-GFAP mouse IgG	Cell signaling	3670	1:400	1:1000
Anti-GFAP rabbit IgG	Abcam	Ab7260	1:400	
Anti-cleaved caspase 3 rabbit IgG	Cell signaling	9664	1:200	
Anti-Aβ (4G8) mouse IgG	Biolegend	800701	1:200	
Anti-Map2 mouse IgG	Millipore	MAB3418	1:200	
Anti-Sox2 rat IgG	Invitrogen	14-9811-82	1:200	1:1000
Anti-Sox2 rabbit IgG	Abcam	Ab97959		1:1000
Anti-Dcx rabbit IgG	Abcam	Ab18723	1:200	1:1000
Anti-NeuN rabbit IgG	Cell signaling	24307	1:300	
Anti-ki67 rabbit IgG	Abcam	Ab15580	1:200	
Anti-Neurod1 mouse IgG	Abcam	Ab60704	1:200	1:1000

Anti-Tau rabbit IgG	Cell signaling	46687	1:200	
Anti-$P15^{Ink4b}$ mouse IgG	Novus	NBP2-45603		1:500
Anti-β-actin mouse IgG	sigma	A5441		1:2000
Anti-GAPDH mouse IgG	Santacruz	Sc32233		1:4000
Anti- α-tubulin mouse IgG	Cell signaling	3873		1:2000
Goat anti-mouse Alexa 488	Thermo Fisher	A21121	1:200	
Goat anti-rabbit Alexa 555	Thermo Fisher	A21428	1:200	
Goat anti-rabbit Alexa 488	Thermo Fisher	A11008	1:200	
Goat anti-rat Alexa 555	Thermo Fisher	A21434	1:200	
Goat anti-mouse Alexa 647	Thermo Fisher	A32728	1:200	
Goat anti-mouse HRP	Thermo Fisher	31430		1:2000
Goat anti-rabbit HRP	Thermo Fisher	31460		1:2000
Goat anti-rat HRP	Thermo Fisher	31470		1:2000

Supplementary Table 3. List of primers for mouse NSCs

Gene	Primers	
Gapdh	Forward	5’-CGTGCCGCCTGGAGAAACC-3’
	Reverse	5’-TGGAAGAGTGGGAGTTGCTGTTG-3’
Tuj1	Forward	5’-GGTCTGGCGCCTTTGGA-3’
	Reverse	5’-CACCACTCTGACCAAAGATA-3’
Cdkn2b (P15)	Forward	5’-ATCCCAACGCCCTGAACCGCT-3’
	Reverse	5’-AGTTGGGTTCTGCTCCGTGGAG-3’
Neurog2	Forward	5’-AGACGGTGCAGCGCATCAAGAA-3’
	Reverse	5’-AGCGTCTCGATCTTCGTGAGCT-3’

Supplementary Table 4. List of primers for human iPSCs derived NSCs

Gene	Primers	
GAPDH	Forward	5’- GCCACATCGCTCAGACACC -3’

	Reverse	5’- AATCCGTTGACTCCGACCTTC -3’
SOX2	Forward	5’- GAGCTTTGCAGGAAGTTTGC -3’
	Reverse	5’- GCAAGAAGCCTCTCCTTGAA -3’
DCX	Forward	5’- CCAAGCCTATCATTGTAGTAG -3’
	Reverse	5’- CAGAGGAGAAATCACAGG -3’
